# Sugar Puckering Drives G‐Quadruplex Refolding: Implications for V‐Shaped Loops

**DOI:** 10.1002/chem.201904044

**Published:** 2019-12-10

**Authors:** Linn Haase, Jonathan Dickerhoff, Klaus Weisz

**Affiliations:** ^1^ Institute of Biochemistry University of Greifswald Felix-Hausdorff-Str. 4 17487 Greifswald Germany; ^2^ Present address: Department of Medicinal Chemistry and Molecular Pharmacology College of Pharmacy Purdue University West Lafayette IN 47907 USA

**Keywords:** glycosidic torsion angles, G-quadruplexes, NMR spectroscopy, sugar conformation, V-loop

## Abstract

A DNA G‐quadruplex adopting a (3+1) hybrid structure was modified in two adjacent *syn* positions of the antiparallel strand with *anti*‐favoring 2′‐deoxy‐2′‐fluoro‐riboguanosine (^F^rG) analogues. The two substitutions promoted a structural rearrangement to a topology with the 5′‐terminal G residue located in the central tetrad and the two modified residues linked by a V‐shaped zero‐nucleotide loop. Strikingly, whereas a sugar pucker in the preferred *north* domain is found for both modified nucleotides, the ^F^rG analogue preceding the V‐loop is forced to adopt the unfavored *syn* conformation in the new quadruplex fold. Apparently, a preferred C3′‐*endo* sugar pucker within the V‐loop architecture outweighs the propensity of the ^F^rG analogue to adopt an *anti* glycosidic conformation. Refolding into a V‐loop topology is likewise observed for a sequence modified at corresponding positions with two riboguanosine substitutions. In contrast, 2′‐F‐arabinoguanosine analogues with their favored *south*‐*east* sugar conformation do not support formation of the V‐loop topology. Examination of known G‐quadruplexes with a V‐shaped loop highlights the critical role of the sugar conformation for this distinct structural motif.

## Graphical abstract

**Refolding of a (3+1) hybrid** G‐quadruplex into a quadruplex with an interrupted G‐tract and a V‐shaped loop is enforced by two 2′‐deoxy‐2′‐fluoro‐riboguanosine substitutions. The analogues flanking the V‐loop in the rearranged structure adopt *syn* and *anti* glycosidic torsion angles and a *north*‐type sugar pucker, the latter being identified as a critical parameter for V‐loop formation.

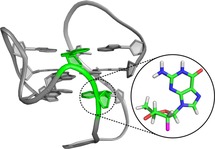

## Introduction

G‐quadruplexes (G4s), formed by the stacking of guanine tetrads with their square‐planar arrangement of hydrogen‐bonded guanine bases, have attracted growing interest in the past years due to their existence and potential regulatory role in vivo.^1^, ^2^, ^3^ The remarkable variety of topologies as displayed by these four‐stranded DNA structures^4^, ^5^ makes them promising tools for various technological applications.^6^, ^7^ G‐quadruplexes can be composed of one (monomolecular), two (bimolecular), three (trimolecular), or four (tetramolecular) strands. Depending on the relative strand orientation as fixed by the type of loops in case of monomolecular G4s, topologies can be of a parallel, antiparallel, or hybrid type. Additional features such as bulges,^8^, ^9^, ^10^ capping structures like base triplets,^11^, ^12^, ^13^, ^14^, ^15^, ^16^, ^17^ interrupted G‐tracts and V‐loops,^11^, ^15^, ^18^, ^19^, ^20^, ^21^, ^22^, ^23^, ^24^ as well as interlocked G4s^18^, ^25^, ^26^ further expand the topological landscape of these highly polymorphic structures. Notably, despite an ever‐growing number of available high‐resolution structures our understanding of the factors driving G4 folding to a particular topology remains limited. Thus, the folding topology and thermal stability strongly depends on the sequence and the length of loops and flanking residues.^14^, ^27^ Also, environmental effects such as buffer pH^12^, ^28^ or the nature of coordinating cations^29^ can all have a significant impact on the G4 conformation, further complicating structural predictions.

**Figure 1:**
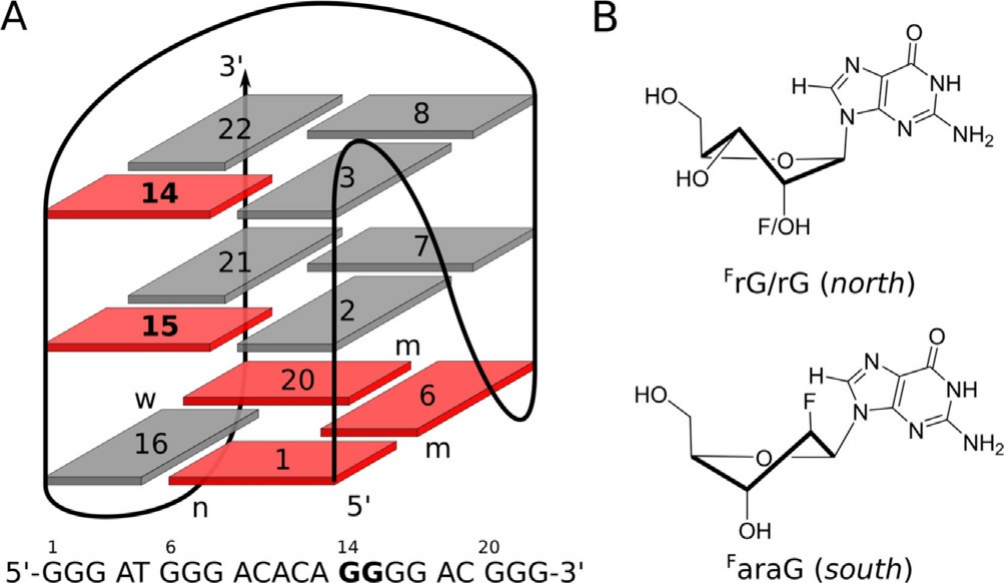
(A) Sequence and schematic representation of the *ODN* G‐quadruplex. *Anti* and *syn* G‐core residues are represented by grey and red rectangles, m, n, and w denote medium, narrow, and wide grooves, respectively. Modification sites are highlighted in bold. (B) *Anti*‐favoring G analogues in their typical sugar conformation; ^F^rG and rG favor a *C3′‐endo* (*north*) sugar pucker whereas ^F^araG has a propensity to adopt a *south*‐type conformation.

Approaches for a versatile and rational G4 design are expected to heavily support many bio‐ and nanotechnological G4 applications. A powerful and efficient strategy for the directed manipulation of the topology for a given G4‐forming sequence involves the deliberate incorporation of G‐analogues.^30^ Here, the glycosidic torsion angle plays a major role as it is correlated with strand orientation and tetrad polarity. Apart from a complete disruption of the quadruplex fold, an *anti* to *syn* or *syn* to *anti* transition is necessarily accompanied by an inversion of either the tetrad polarity or the G‐tract orientation. Thus, 8‐substituted G analogues such as 8‐methyl‐ or 8‐bromo‐2′‐deoxyguanosine have been used to drive *anti*→*syn* transitions, often causing a tetrad polarity reversal when incorporated into the 5′‐tetrad of parallel G4 structures.^31^, ^32^, ^33^, ^34^ Likewise, some 2′‐substituted G surrogates can be used to enforce *anti* glycosidic conformations (Figure 1 B). 2′‐Deoxy‐2′‐fluoro‐riboguanosine (^F^rG) and riboguanosine (rG) prefer a *C3′‐endo* sugar pucker thought to be strongly correlated with an *anti* conformation. Another *north*‐favoring G analogue with a strong propensity for the *anti* glycosidic torsion angle is the bicyclic 2′‐*O*‐4′‐*C*‐methylene rG analogue (locked nucleic acid, ^LNA^G), which is strictly locked in a *C3′‐endo* conformation. In contrast, 2′‐deoxy‐2′‐fluoro‐arabinoguanosine (^F^araG) has an *anti* preference but a tendency to adopt a sugar pucker in the *south‐east* pseudorotational domain.

Recently, 2′‐substituted G analogues have been incorporated at various sites into a (3+1)‐hybrid quadruplex termed *ODN* featuring a propeller, diagonal, and lateral loop (Figure 1 A).^35^ Substituting two or three *syn*‐residues in the 5′‐tetrad with ^F^rG, rG, or ^F^araG caused a reversal of the tetrad polarity under conservation of the global quadruplex fold.^36^, ^37^, ^38^, ^39^ On the other hand, incorporating ^F^rG or ^F^araG into the single *syn*‐position of the central tetrad lead to partial refolding into an antiparallel topology associated with an inversion of the first G‐tract and of the central tetrad polarity.^40^ In both cases, *anti*‐favoring G analogues had very similar overall effects and differences were only observed in relative thermal stabilities and local structure, e.g., position‐dependent sugar conformations and participation of the 2′‐substituent in pseudo‐hydrogen bonds.

Here, we expand our studies on specific *ODN* modifications by evaluating dual ^F^rG substitutions at the two *syn*‐positions of its antiparallel G‐tract. These modifications are found to promote an unexpected refolding into a well‐defined topology with a V‐shaped loop connecting the two ^F^rG residues. A detailed conformational analysis of the V‐loop supporting G analogues and additional modifications including rG and ^F^araG reveals that the preferred sugar pucker outweighs glycosidic torsion angle propensities in constituting the major driving force for V‐loop formation.

## Results

### Resonance assignments for the ^F^rG‐modified ODN

**Figure 2:**
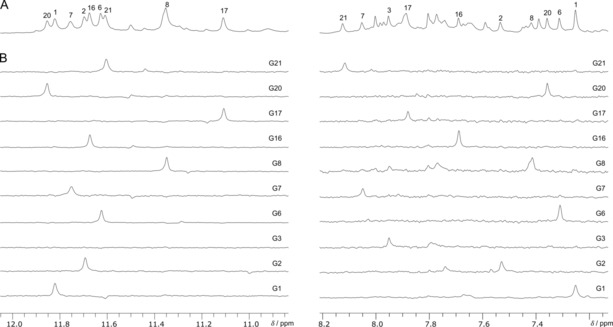
Assignment of H1 (left) and H8 resonances (right) through ^15^N filtered HMQC experiments of individually ^15^N labeled samples (5–10 % ^15^N). One‐dimensional ^1^H (A) and ^1^H–^15^N HMQC spectra (B) were acquired at 25 °C with a typical concentration of 0.2 mm in 10 mm KP_i_ buffer at pH 7.

Initially, the *ODN* sequence was modified with two ^F^rG substitutions at position 14 and 15 and analyzed by 1D ^1^H NMR spectroscopy. This F(14,15) variant folds into a major G‐quadruplex as indicated by the presence of at least ten well resolved imino resonances in the 10.8–12.0 ppm region typical for Hoogsteen hydrogen bonding (Figure 2 A). Individual ^15^N labeling of all G residues except for G22 at the 3′‐terminus and the two ^F^rG analogues was used for the assignment of both guanine imino and H8 resonances through spectral editing with one‐dimensional ^1^H–^15^N HMQC experiments (Figure 2 B). Surprisingly, G17 being a loop residue in the native structure participates in G‐core formation of the new fold as clearly demonstrated by its imino signal at 11.1 ppm. In contrast, G3 does not show any imino resonance and is thus identified as a loop residue. Assignments of H8 and H1 resonances were additionally validated by their intra‐base correlation to ^13^C5 as observed in a ^1^H–^13^C HMBC experiment (Figure S1). Sequential NOE contacts can be traced between all unmodified Gs and NOE walks further extend into the short intervening sequences following and preceding the terminal G‐tracts (Figure S2). These allowed for the assignment of non‐exchangeable protons of unlabeled G22 at the 3′‐end of the sequence as well as of loop residues A4, T5, A18, and C19. G1, G6, and G20 were identified as *syn* residues by their strong H8–H1′ intranucleotide NOE crosspeak and their characteristic downfield ^13^C8 chemical shift (Figure S2 and S3). H1′ and H2′ resonances of ^F^rG can easily be detected by their characteristic ^1^H–^19^F scalar coupling to F2′ (see below). It should be noted, however, that only one ^F^rG residue shows up at 25 °C with resonances of the other analogue apparently broadened beyond detection at this temperature, also explaining the reduced number of imino signals (Figure 2 A).

### F(14,15) adopts a V‐loop topology

**Figure 3:**
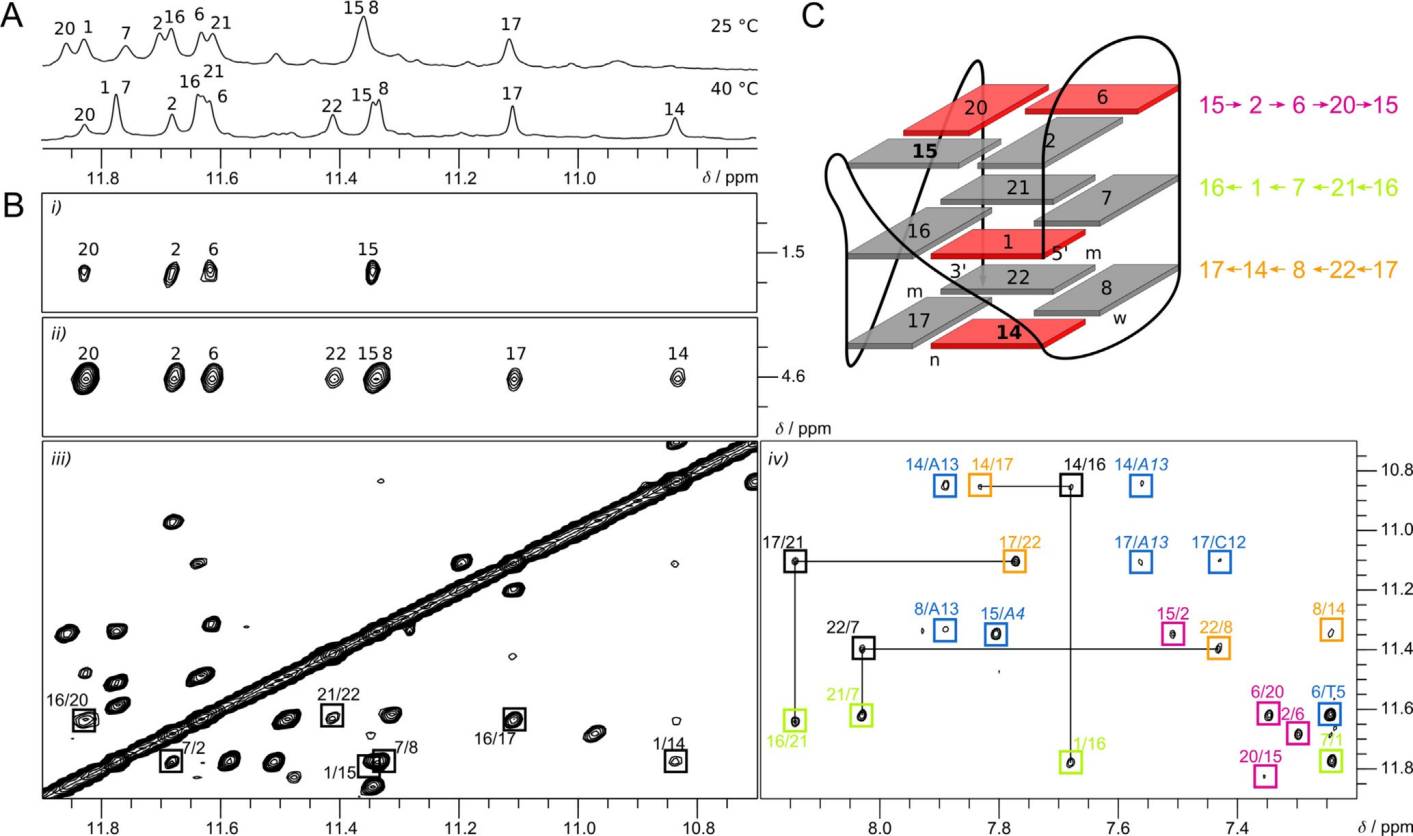
Determination of the F(14,15) topology. (A) Imino region of 1D ^1^H NMR spectra at 25 °C and 40 °C. (B) Portions of 2D NOE spectra of F(14,15) (1 mm) at 40 °C in 10 mm KP_i_, pH 7. *i)* NOE contacts of the four imino protons in the top tetrad to T5 Me in the first lateral loop. *ii)* Exchange crosspeaks between water and imino protons of the outer tetrads. *iii)* Inter‐tetrad H1‐H1 contacts, reflecting relative tetrad polarities (marked by squares). Note additional exchange crosspeaks due to minor species. *iv)* H1(ω_1_)–H8(ω_2_) contacts, reflecting hydrogen bond directionalities within tetrads (magenta, green, and orange squares for top, central, and bottom tetrad, respectively), NOE contacts between tetrads (black squares traced by horizontal and vertical lines), and NOE contacts between outer tetrad imino resonances and base protons of residues in the two lateral loops, namely A4, T5, A13, and C12 (blue squares). Adenosine H2 resonances are labeled in italic. (C) Folding topology of F(14,15). *Anti* and *syn* residues are shown in grey and red; m, n, and w denote medium, narrow, and wide grooves, respectively.

Upon increasing the temperature to 40 °C, sugar and H8 resonances of the second ^F^rG resonance are clearly observable (Figure S3 and S4). Also, an additional upfield shifted imino resonance emerges at 10.84 ppm and another slightly broadened signal at 11.41 ppm becomes resolved (Figure 3 A). Supported by completed assignments at 25 °C and only minor changes of the crosspeak patterns in 2D NOE and ^1^H–^13^C HSQC spectra at 40 °C (Figure S3 and S4), non‐exchangeable base and sugar resonances were likewise assigned through sequential NOE contacts at the elevated temperature. Also, correlations with H8 protons through ^13^C5 in a ^1^H–^13^C HMBC experiment allowed for the unambiguous assignment of H1 resonances at 40 °C (Figure S5). Thus, the two newly emerging imino signals were easily allocated to G22 and ^F^rG14. Surprisingly, the latter adopts the unfavored *syn* conformation as indicated by its downfield ^13^C8 chemical shift and by its weak H8–H2′ and strong H8–H1′ intra‐residual NOE contact (Figure S3 and S4). Unfortunately, with only a modest thermal stability in the buffer used for the NMR experiments (Table S1), the structure is partially unfolded at elevated temperatures as reflected in some poorly dispersed peaks from single‐stranded species in the ^1^H–^13^C HSQC spectrum (Figure S3). Nevertheless, NMR experiments on samples in a low‐salt buffer at higher temperatures provided the best spectral quality and were therefore selected for a more detailed structural characterization (see also Figure S6).

The quadruplex topology as depicted in Figure 3 C is unambiguously established through H8‐H1 NOE contacts within the tetrads, showing the following hydrogen bond directionalities: 2→6→20→15; 1→16→21→7; 14→17→22→8 (Figure 3 B). G1 is placed into the central tetrad and a zero‐nucleotide V‐shaped loop connects ^F^rG14, located in the bottom tetrad to occupy the free position within the first G‐tract, and ^F^rG15, located in the top tetrad within the third G‐tract. Relative tetrad orientations result in both heteropolar and homopolar stacking interactions in line with H1–H1 NOE contacts between and within G‐tracts, respectively. Of note, strand inversion occurs within the third G‐tract between ^F^rG15 and G16. Also, NOE contacts between T5 Me within the first lateral loop and the imino resonances of G2, G6, G20, and ^F^rG15 residues in the top tetrad are conspicuous. Together with imino signals of four other residues, namely G8, G17, G22, and the ^F^rG14 analogue, the same four imino signals exhibit exchange peaks with water due to their location within outer tetrads and their faster solvent exchange.

### V‐loop supporting ^F^rG analogues adopt an *N*‐type sugar conformation

**Figure 4:**
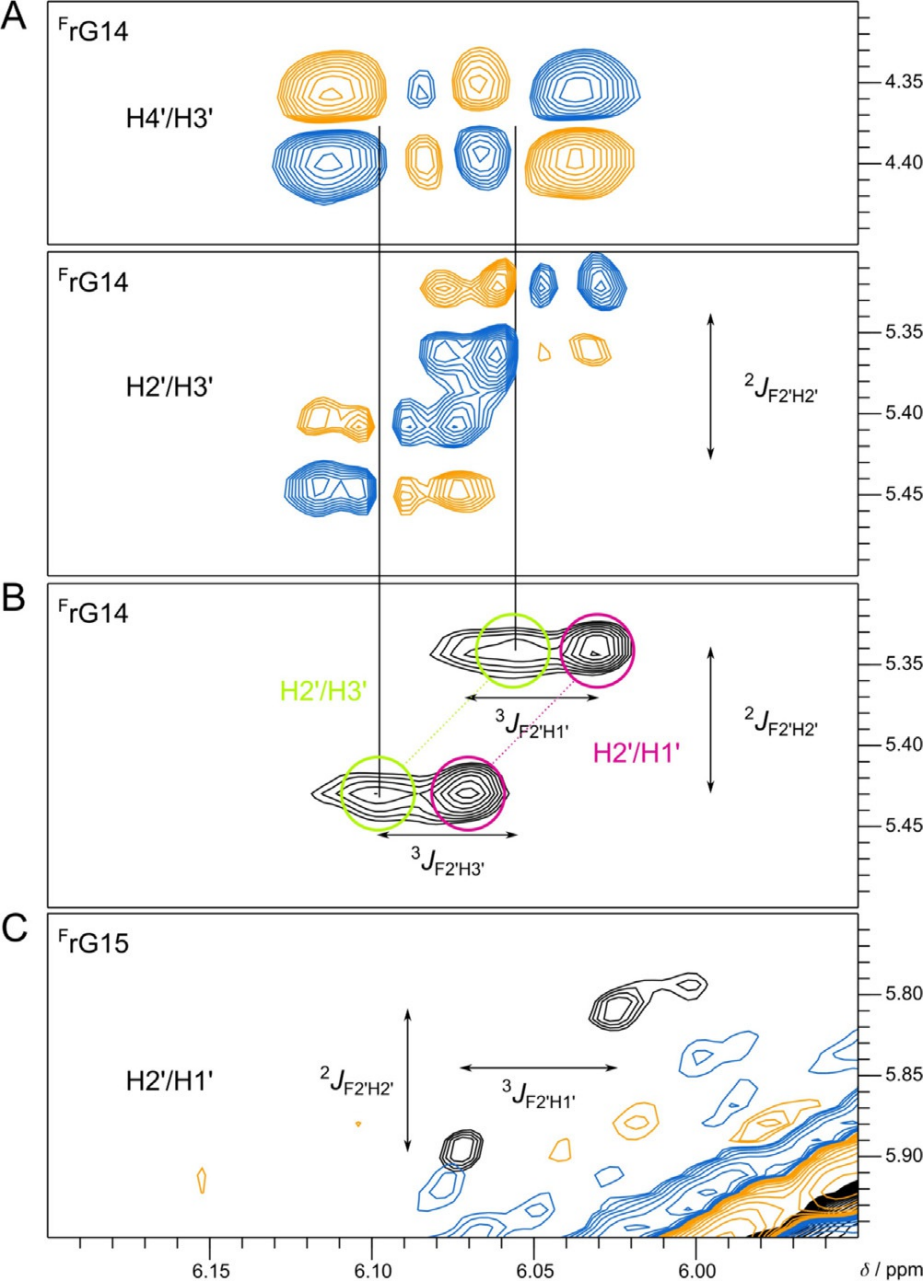
Portions of DQF‐COSY and 2D NOE spectra of F(14,15) (1 mm) at 40 °C in 10 mm KP_i_, pH 7. (A) H4′(ω_1_)–H3′(ω_2_) and H2′(ω_1_)–H3′(ω_2_) DQF‐COSY crosspeaks of ^F^rG14. (B) H2′(ω_1_)–H1′/H3′(ω_2_) NOE contacts for ^F^rG14. (C) Superimposed ^F^rG15 H2′(ω_1_)–H1′(ω_2_) spectral region of a 2D NOE (black) and DQF‐COSY spectrum (colored) only showing an NOE crosspeak. While the nearly isochronous H3′ and H1′ resonances of ^F^rG14 exhibit NOE contacts to H2′, only H3′ shows an observable DQF‐COSY crosspeak to H2′, pointing to a *north*‐type sugar pucker. The H4′–H3′ DQF‐COSY crosspeak confirms the assignment of the downfield shifted H3′ resonance. Splittings due to passive ^19^F–^1^H scalar couplings are indicated.

Although ^F^rG14 is forced into an unfavored *syn* conformation, refolding of modified *ODN* is apparently driven by the two fluorine‐substituted analogues being anchor points for the newly formed V‐loop. In an attempt to better understand the driving force for such a rearrangement and to evaluate V‐loop conformational features, sugar conformations of the two ^F^rG residues were subjected to a more detailed analysis. Conspicuously, H1′–H2′ crosspeaks are clearly observable in 2D NOE but unobservable in DQF‐COSY spectra for both residues, pointing to small ^3^ *J*_H1′H2′_ scalar couplings with cancellation of antiphase COSY crosspeaks and sugar puckers in the *north* domain of the pseudorotation cycle (Figure 4). Facilitated by the E.COSY‐type pattern of H1′‐H2′ and H2′‐H3′ crosspeaks as a result of additional ^19^F passive couplings, ^3^ *J*_F2′H1′_ and ^3^ *J*_F2′H3′_ could be directly extracted from corresponding 2D NOE and DQF‐COSY correlations without resorting to simulations. Also, following the unambiguous assignment of ^19^F resonances to ^F^rG14 and ^F^rG15 in a HOESY experiment through heteronuclear ^19^F‐^1^H dipolar couplings (Figure S7), ^3^ *J*_HF_ scalar couplings were independently determined by the selective ^1^H decoupling of ^19^F spectra (Figure S8). Of note, the rather unusual conformation of ^F^rG14, combining a *syn* glycosidic torsion angle with a *north* sugar pucker, is associated with an extremely downfield shifted H3′ resonance almost isochronous with its H1′ resonance at 6.05 ppm (see below), preventing the extraction of reliable ^3^ *J*_HF_ couplings in ^F^rG14 from selective decoupling experiments. Using the observed ^19^F–^1^H scalar couplings (Table S2) together with a Karplus‐type relationship between vicinal ^1^H–^19^F coupling constants and H‐C−C‐F torsion angles for the 2′‐fluoro sugar,^41^ major pseudorotamers of both analogues were found to adopt a *north‐east* pucker with a phase angle of pseudorotation 40° < *P*<70°.

### Three‐dimensional structure of F(14,15)

For a deeper structural insight, we determined the high‐resolution structure of the modified F(14,15) quadruplex from NMR‐derived distance and torsion angle restraints. Whereas A9 H8 could be assigned based on sequential NOE contacts to G8 (Figure S5), other adenosine and cytidine residues of the long and flexible 5‐nt lateral loop preceding the V‐loop escaped their unambiguous identification. In trying to complement assignments, corresponding nucleotides were individually labeled by ^15^N‐dA and 5‐methyl‐dC. Thus, C10 and C12 were easily identified by the disappearance of the corresponding H5 resonance and a shift of H6 and ^13^C6 resonances in the 5‐methyl‐dC modified samples (Figure S9). Also, ^15^N edited spectra of ^15^N‐dA labeled samples allowed for a straightforward assignment of A13 H8 and H2 resonances at 7.89 and 7.56 ppm, respectively, both exhibiting several NOE contacts to imino protons of the bottom tetrad and providing valuable restraints for subsequent structure calculations (Figure 3 B). Unfortunately, assignment of H2/H8 resonances for residue A11 was hampered by intermediate exchange processes at 25 °C and signal overlap with unfolded species at 40 °C (Figure S10).

**Figure 5:**
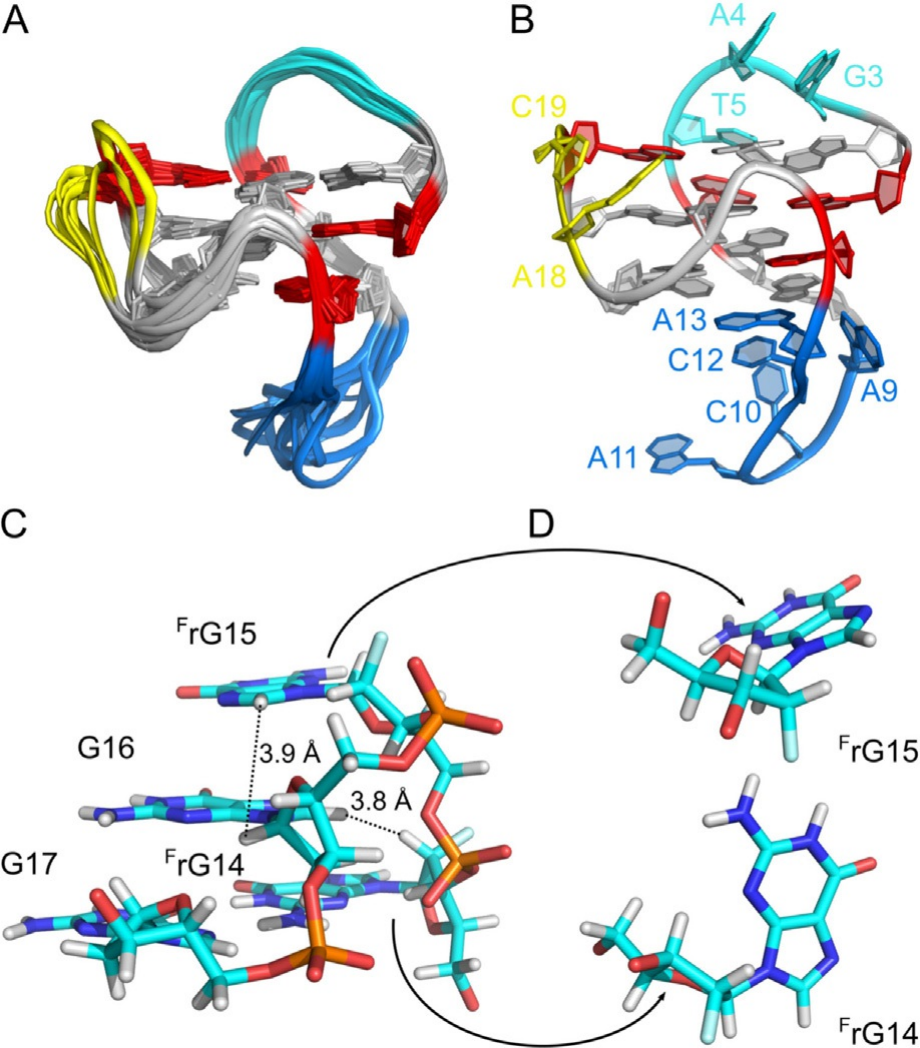
Three‐dimensional structure of F(14,15). (A) Superposition of 10 final low‐energy structures in a sideview with the V‐loop in front. For loop residues only the backbone is shown. (B) Representative structure with labeled loop residues. G‐core *syn* residues are colored in red; the first and second lateral loop and the propeller loop are shown in cyan, blue, and yellow, respectively. (C) Detailed view on the V‐loop and the third G‐tract of a representative structure in stick representation. Short interproton distances between ^F^rG15 H8 and G16 H1′ as well as between G16 H8 and ^F^rG14 H2′ are traced by broken lines. (D) Stick representation of *anti* ^F^rG15 and *syn* ^F^rG14, both adopting a *C4′‐exo* conformation. In the latter, H3′ is positioned in close proximity to the 6‐membered ring of the guanine base, rationalizing its downfield chemical shift.

A superposition of ten final structures determined by molecular dynamics calculations in explicit water (Table S3) together with a representative structure is shown in Figure 5 A and B. RMSD values of 0.84 Å and 2.96 Å for the G‐core and the overall structure are similar to values reported for the unmodified (3+1) hybrid G4.^35^ All G‐core residues are well defined, including the 5′‐terminal guanosine in the central tetrad as well as the two V‐loop flanking G analogues. While the first lateral loop is well structured with G3 and T5 loosely stacked onto the upper tetrad, the other two loops experience higher flexibility. In particular, C19 within the 2‐nt propeller loop as well as C10 and A11 in central positions of the long 5‐nt lateral loop are very dynamic. In contrast, the two lateral loop residues directly preceding the V‐loop are well defined with A13 stacked onto the bottom tetrad.

The structure features two medium grooves, one wide groove bridged by two lateral loops at either side, and a narrow groove spanned by the 0‐nt V‐loop (Figure 3 C). A peculiarity derives from the ^F^rG15 analogue located at the V‐loop 3′‐end and being part of the third G‐column. By adopting a favored *anti* glycosidic torsion angle, its opposite base orientation when compared to the following *anti*‐G16 demands inversion of the 5′→3′ strand orientation between the two residues within the same G‐tract.^5^ This is recognized by the sharp turn of the sugar‐phosphate backbone between ^F^rG15 and G16 and seems to rely on a C4′*‐exo* sugar pucker of ^F^rG15 (Figure 5 C). Notably, the *anti* conformation of this G‐column 5′‐residue as a consequence of its flipped backbone orientation is fully compatible with the narrow groove geometry between the first and third G‐tract.

G‐core residues in a *syn* conformation include G1, G6, G20, as well as ^F^rG14 which supports the V‐loop at its 5′‐end. The latter adopts this disfavored conformation in spite of its known *anti* preference (Figure 5 D). The *north*‐type sugar conformation in combination with a *syn* glycosidic torsion angle positions the ^F^rG14 H3′ proton in close proximity to the deshielding region of its guanine base, accounting for its unusual downfield shift (Figure 5 D). Also, the non‐conventional (H8_*i*_–H2′_*i*−2_) contact between G16 and ^F^rG14 is reflected in the 3D structure with corresponding distances in the range 2.8–3.9 Å (Figure 5 C). As another consequence of the *north* sugar, both fluorine atoms are oriented away from the quadruplex narrow groove in F(14,15). Avoiding the positioning of 2′‐substituents within a G4 narrow groove has been observed before and attributed to unfavorable interactions.^37^, ^39^ Of note, the two F2′ substituents of the V‐loop flanking residues are not involved in any pseudo‐hydrogen bond as has frequently been observed in ^F^rG, but also ^F^araG and rG modified G4 structures.^37^, ^38^, ^39^, ^40^, ^42^, ^43^

### Structural impact of other modifications at position 14 and 15: Importance of the sugar pucker

**Figure 6:**
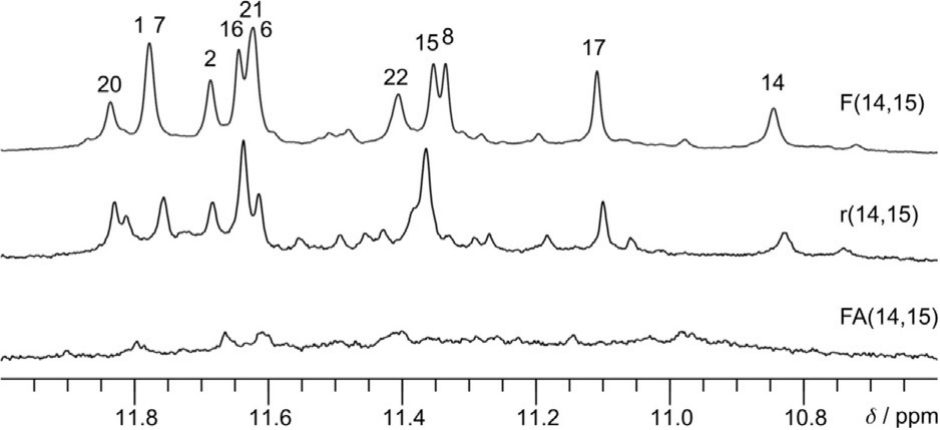
Imino proton spectral region of F(14,15), r(14,15), and FA(14,15) at 35 °C (10 mm KPi buffer, pH 7).

To more generally assess the role of a *north* sugar pucker for V‐loop formation, we studied additional 14,15‐disubstituted *ODN* quadruplexes. Thus, sequences r(14,15) and FA(14,15) were modified with two riboguanosines and two 2′‐fluoro‐2′‐arabinoguanosines, respectively. Whereas rG favors *north* conformers, the ^F^araG analogue has a propensity for a *south‐east* sugar pucker. Just like F(14,15), both sequences exhibit a CD signature typical of a hybrid‐type G4 with both homopolar and heteropolar stacking interactions (Figure S11). However, the imino proton spectral region of FA(14,15) revealed that it does not fold into a major quadruplex species, suggesting a detrimental effect for folding into either the native or the rearranged V‐loop structure of the two ^F^araG substitutions with their favored *south*‐*east* sugar pucker and *anti* conformation (Figure 6). In fact, ^19^F NMR spectra indicate a polymorphic mixture with several coexisting species (Figure S12). In contrast, imino resonances for r(14,15) clearly point to a major folded species with a pattern of imino signals similar to F(14,15). ^1^H–^13^C HSQC spectra of r(14,15) show an almost perfect overlap with corresponding spectral regions of F(14,15) (Figure S13), suggesting that they share the same topology and allowing for the assignment of most r(14,15) resonances. Sequential contacts can be traced along all G‐tracts and along the first lateral loop. Analogous to F(14,15), G1, G6, G20, as well as modified rG14 can be identified as *syn* residues from their downfield ^13^C8 chemical shift and their strong intra‐residual H8–H1′ NOE contact (Figure S13 and S14). In addition, H1–H1 and H8–H1 NOE contacts are in full agreement with a V‐loop topology and confirm folding into the same G4 structure (Figure S15).

Conformational features of the rG nucleosides in r(14,15) are very similar to the 2′‐fluoro‐G analogues in F(14,15). In both G4s, the modified residues adopt a *north*‐type sugar pucker as revealed by the presence and absence of H1′–H2′ correlations in a 2D NOE and DQF‐COSY spectrum, respectively (see Figure S16). A particularly strong H1′–H4′ contact points to a *north‐east* pucker for rG15. Remarkably, the (*syn*,*north*) conformation of residue 14 is very similar among the two structures as shown by unusually downfield shifted H3′ resonances at 5.84 ppm for r(14,15). Again, the deshielding of H3′ can be attributed to its positioning close to and almost in plane with the guanine base (see also Figure 5 D). Other diagnostic interactions sensitive to the V‐loop conformation involve the H8_*i*_–H2′_*i*−2_ NOE contact between G16 and rG14 and an unusual sequential NOE contact between rG H8 at position 15 and G16 H1′ (see Figure 5 C and S17). It should be noted, however, that the latter H8_*i*_–H1′_*i*+1_ NOE crosspeak escapes unambiguous identification in F(14,15) due to nearly isochronous H1′ resonances of G16 and ^F^rG15.

Taken together, dual modifications with *south‐east* favoring ^F^araG analogues seem incompatible with V‐loop formation of the *ODN* sequence. In contrast, *north* favoring G substitutes induce rearrangements into the same well defined V‐loop structure termed ODN(14,15) for the following discussion.

## Discussion

A V‐shaped loop was first reported for a two‐layered G‐quadruplex forming an interlocked dimer.^18^ In the past years, a growing number of such V‐shaped loops have been found in various three‐layered bi‐ or monomolecular G4s, making the V‐loop a more recurrent structural motif in G‐quadruplexes.^11^, ^15^, ^19^, ^20^, ^21^, ^22^, ^23^, ^24^ It links two adjacent antiparallel G‐columns through residues located on opposite outer faces of the G‐core. Starting with a guanosine in the bottom tetrad of a broken G‐tract it is easily distinguished from a propeller‐type loop by the upward orientation of this G nucleotide at the V‐loop 5′‐end (Figure S18).

Of note, the particular V‐loop topology adopted by ODN(14,15) with two lateral loops followed by a V‐loop and a propeller loop was also reported for *CHL1* and *HPV52* sequences from the 5′‐intron of the human *CHL1* gene and the G‐rich region of the human papillomavirus type 52.^11^, ^21^, ^22^ Interestingly, *HPV52* and *ODN* are derived from the same sequence encompassing five G‐runs. Whereas *HPV52* comprises G‐tracts I‐IV, the *ODN* sequence includes G‐tracts II‐V with additional inversion of the 5′‐3′ orientation.^21^ ODN(14,15), *HPV52*, and *CHL1* differ in the length and sequence of loops, but they all feature a lateral loop adenosine directly preceding the V‐loop. Also, the V‐loop 3′‐supporting G residue adopts an *anti* glycosidic conformation while a *syn* G anchors the V‐loop at its 5′‐end in all structures.

Only considering glycosidic torsion angle propensities of the introduced *anti*‐favoring ^F^rG surrogates, a V‐loop structure is anticipated to be favored with respect to the native (3+1) hybrid fold with *syn* conformers at both modification sites. Yet, the observed rearrangement of modified ODN(14,15) into the V‐loop quadruplex with its single *syn* ^F^rG analogue is not easy to account for. In fact, a *syn* conformation is highly unusual for ^F^rG nucleotides but not without precedence. Thus, a *syn* ^F^rG residue was found in the native fold of a 15‐^F^rG mono‐substituted *ODN* sequence F(15) (see Figure 1 A) that coexists with another rearranged topology comprising ^F^rG in an *anti* conformation.^40^ Likewise, a *syn* rG in r(14,15) seems highly unfavorable considering RNA′s preference for an *anti* glycosidic torsion angle. In fact, rGs in the *syn* conformation have rarely been reported with a few notable exceptions in non‐parallel G4 structures^44^, ^45^, ^46^ and are otherwise primarily known from Z‐RNA.^47^ This suggests, that it is the propensity for a *north*‐type sugar pucker rather than glycosidic torsion angle preferences of the modified nucleosides that critically determines the folding of ODN(14,15). Accordingly, ^F^araG substitutions favoring a *south*‐*east* sugar pucker seem to disrupt the native fold without promoting V‐loop formation. This is in striking contrast to previously studied quadruplexes, where ^F^rG and ^F^araG analogues showed similar effects when incorporated at corresponding positions.^38^, ^40^, ^43^, ^48^, ^49^ Notably, ^F^araG modifications imparted higher thermal stabilities to most of the structures and were more effective in inducing rearrangements to a new topology or in the selection of a particular G4 conformation. Consequently, observed substitution effects and conformational details of the ODN(14,15) quadruplex indicate that mostly *north* pseudorotamers are compatible with its 0‐nt V‐loop geometry, which is likely restricted to a narrow conformational range of backbone torsion angles.

**Figure 7:**
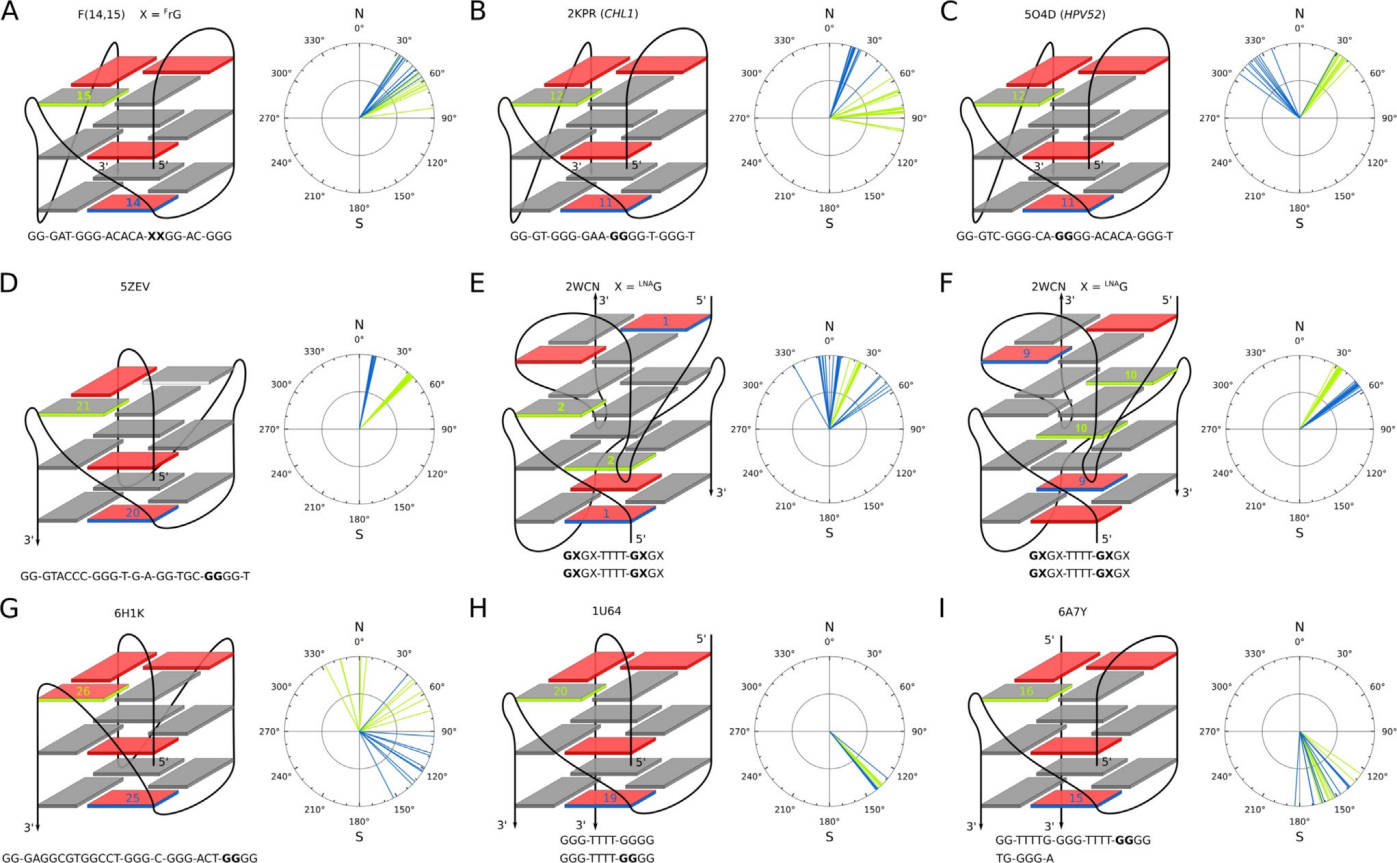
Sugar pucker analysis of V‐loop flanking residues in F(14,15) and in published high‐resolution G4 structures. Quadruplexes are shown in a schematic representation with *anti* and *syn* G‐core residues represented by grey and red rectangles. These include (A) F(14,15), (B) *CHL1*,^11^ (C) *HPV52*,^22^ (D) a monomolecular G4 formed by a sequence derived from the *VEGFR‐2* promoter,^15^ (E,F) an LNA modified bimolecular G4,^50^ (G) a monomolecular G4 formed by a G‐rich HIV‐1 long terminal repeat sequence,^23^ (H) a bimolecular G4 formed by d[G_3_T_4_G_4_]_2_,^19^ (I) an asymmetric bimolecular G4 composed of one short and one long G‐rich strand.^24^ V‐loop flanking residues are labeled with residue numbers. The pseudorotation angle found for the residues preceding (blue) and following the V‐loop (green) is plotted for individual low energy structures. PDB accession codes and sequences are indicated.

Perusal of all G4s with V‐shaped loops that have been reported to date reveals a pronounced prevalence of *north‐east* pseudorotamers within the V‐loop linked segment for the monomolecular quadruplexes (Figure 7 A–D, G). The *HPV52* G4 features a pseudorotation angle for the *syn* residue preceding the V‐shaped loop of about 320°, being outside the typical *north* pseudorotational range and even more remote from a *C2′‐endo* pucker usually favored by the G nucleotide (Figure 7 C).^22^ However, the conformation of this residue in *HPV52* and ODN(14,15) is very similar as demonstrated by the H3′ proton which is unusually downfield shifted in both cases due to its close proximity to the guanine aromatic ring. *North* sugars are also present in a bimolecular V‐loop structure formed by an LNA modified sequence, where the transition from the native antiparallel to a V‐loop scaffold is driven by a locked ^LNA^G modification fixing the V‐loop at its 3′‐end (Figure 7 E, F).^50^ Because ^LNA^G is strictly locked in a *C3′‐endo* conformation it again corroborates a favorable conformational match of a *north*‐type sugar pucker with the V‐loop architecture. Whereas in the latter case a single modification at the V‐loop 3′‐end suffices for a corresponding refolding, this does not apply for the single ^F^rG modification in ODN at position 15,^40^ also pointing to a significant role of a *north*‐type pucker for the V‐loop preceding residue at position 14 in ODN(14,15). In fact, additional 2D NOE spectra acquired on a 14‐^F^rG mono‐substituted *ODN* sequence F(14) showed a number of crosspeaks almost perfectly superimposable with some strong NOE contacts observed for F(14,15) (Figure S19). This suggests its partial folding into a V‐loop architecture even in the absence of a second G analogue at the V‐loop 3′‐flanking position. However, weak signals indicate folding to only occur to a small extent and emphasize the impact and possibly synergistic effect of both consecutive G surrogates in V‐loop formation.

Notable exceptions to the *north‐east* sugar conformation typically associated with V‐shaped loops relate to (i) a monomolecular quadruplex formed by a G‐rich HIV‐1 long terminal repeat sequence, exhibiting a *south* sugar for the V‐loop 5′‐flanking *syn* guanosine (Figure 7 G)^23^ and to (ii) two bimolecular G4s with experimentally determined *south* sugars at both flanking residues (Figure 7 H,I).^19^, ^24^ Remarkably, the former G4 exhibits a different type of V‐loop conformation with a turn of the sugar phosphate backbone occurring between its two V‐loop linked residues rather than within the following G‐tract. As a consequence, the *syn* glycosidic torsion angle at the 3′‐flanking guanosine follows standard rules that link *syn*/*anti* patterns to G‐tract directionalities (Figure S18 B and C). In case of the bimolecular G4s, *C2′‐endo* sugar puckers for the two consecutive Gs in the linked segment can be rationalized by a potentially less constrained V‐loop. Also, glycosidic torsion angles for the V‐loop 3′‐anchoring residue in both G4s are found in the far *high‐anti* region, likely causing a slightly different backbone conformation.

Typical (*north*,*syn*) conformers as observed for the V‐loop 5′‐flanking G residue in most structures seem to violate general beliefs on nucleotide conformational preferences. In fact, successfully shifting equilibria between coexisting G4 topologies by introducing various G analogues is mostly based on a strong correlation between sugar pucker and glycosidic torsion angle. Thus, *C3′‐endo* puckering is often associated with an *anti* glycosidic conformation, attributable to unfavorable steric interactions of a *syn* nucleobase in a pseudoaxial position. In contrast, quantum‐mechanical calculations have determined similar energies for (*north*,*syn*) and (*south*,*syn*) conformers of free deoxyribonucleosides, suggesting that conformational propensities and even corresponding correlations may critically depend on the particular structural context.^51^ Notably, ^F^rG and rG analogues incorporated into a DNA G‐quadruplex have previously been found to enforce a structural rearrangement by adopting an *anti* glycosidic torsion angle, yet with a *C2′‐endo* rather than a favored *C3′‐endo* sugar pucker.^37^, ^38^, ^39^, ^40^ Here, ODN(14,15) exemplifies the reverse situation: Selection for the most favorable *north*‐type sugar pucker apparently outweighs energetic penalties expected for an associated *syn* conformation at the V‐loop 5′‐flanking residue. In this respect, the very malleable *ODN* sequence has proven a powerful tool in studying the impact of 2′‐substituted G modifications in different conformational and topological environments. Depending on the deliberate selection of substitution sites the introduction of G surrogates enabled its refolding into different quadruplexes including (i) a 5′‐tetrad polarity inversion upon conservation of the global native fold,^36^, ^37^, ^38^, ^39^ (ii) a combined G‐tract and tetrad flip resulting in an antiparallel G4,^40^ and (iii) the here reported rearrangement to a V‐loop structure.

While substitution‐induced rearrangements and available V‐loop structures suggest the sugar conformation to play a critical role in V‐loop stabilization, capping structures like base triplets and stable GNA loops have also been recognized as stabilizing elements for individual V‐loop forming sequences. Thus, both *CHL1* and *HPV52* sharing the same topology with ODN(14,15) feature a 3′‐terminal thymidine involved in a base triplet with residues of the second lateral loop that stacks onto the bottom tetrad.^11^, ^22^ Accordingly, the relatively low thermal stability of the ^F^rG and rG modified ODN(14,15) quadruplexes may partially be attributed to the lack of such additional interactions. Combining capping structures and favorable GNA loops with *north*‐affine G‐analogues at appropriate positions may allow the design of sequences adopting very stable V‐loop architectures. This could be of interest for a number of technological applications, as the V‐loop represents a very unique G4 structural element and may serve as a specific receptor for various interacting ligands.

## Conclusions

The growing number of reported G‐quadruplexes featuring a V‐shaped loop attests to their potential significance as important structural elements in vivo but also as powerful tools for technological applications that are based on quadruplex recognition. Disregarding sequence requirements, the V‐loop motif is shown here to be generally supported by two V‐loop flanking *north*‐type conformers, allowing for a more rational design of these structural elements. Remarkably, the sugar pucker seems to constitute the major contributor for V‐loop formation, overwriting glycosidic preferences of the corresponding residues. While glycosidic torsion angle propensities of G analogues have frequently been exploited in the past for a directed modulation of G4 stabilities, the deliberate use of G surrogates for their favored sugar pucker has rarely been employed in the manipulation of G‐quadruplex structures. Clearly, simple rules assist in predicting glycosidic conformations for a particular G4 topology but sugar conformational properties are often neglected and more difficult to predict within a given structural context. In addition, propensities of most G analogues for a particular sugar pucker but also for a glycosidic bond angle are far from being fixed and may depend on particular topological features. As a consequence, the evaluation of modification effects may be challenging in some cases and may set limits to the rational design of G4 structures induced by appropriate G surrogates. On the other hand, many G analogues like ^F^rG and rG may adopt a wide range of conformations to finally stabilize distinct structural motifs, making them an even more powerful tool for a targeted G‐quadruplex design.

## Experimental Section

### Materials and sample preparation

Unlabeled and isotope‐labeled ^F^rG, rG, ^F^araG, and 5‐methyl‐dC modified oligonucleotides were purchased from IBA (Göttingen, Germany) or Microsynth (Balgach, Switzerland) and quantified based on their absorbance at 260 nm after ethanol precipitation. NMR samples were prepared by dissolving the corresponding oligonucleotide in 10 mm potassium phosphate buffer at pH 7, followed by heating to 80 °C and cooling to room temperature. Concentrations for unlabeled NMR samples ranged between 0.1 mm for F(14) and 1 mm for F(14,15) and for ^15^N or 5‐methyl‐dC labeled F(14,15) samples between 0.2 mm and 0.3 mm. For optical measurements, oligonucleotide concentrations of 5 μm were used in a buffer containing 20 mm potassium phosphate, 100 mm KCl, pH 7.

### Optical measurements

Circular dichroism (CD) spectra were acquired with 5 accumulations, a scanning speed of 50 nm min^−1^ and a bandwidth of 1 nm at 35 °C on a Jasco J‐810 spectropolarimeter (Jasco, Tokyo, Japan). All spectra were blank‐corrected by subtraction of the buffer spectrum. Melting curves were recorded in triplicate on a Cary 100 spectrophotometer equipped with a Peltier temperature control unit (Varian Deutschland, Darmstadt) with quartz cuvettes of 10 mm path length. The absorbance at 295 nm was measured between 15 and 90 °C in 0.5 °C intervals with a heating rate of 0.2 °C min^−1^. The melting point *T*_m_ was determined from the minimum of the first derivative of the heating phase.

### NMR spectroscopy

All NMR spectra were acquired on a Bruker Avance Neo 600 MHz spectrometer equipped with an inverse ^1^H/^13^C/^15^N/^19^F quadruple resonance cryoprobehead and *z*‐field gradients. For spectral processing and analysis, Topspin 4.0.4 and CcpNmr Analysis 2.4 were employed.^52^ Further experimental details are given in the Supporting Information.

### Structure refinement

A simulated annealing protocol in Xplor‐NIH 2.49 was used to generate 100 starting structures of the DNA sequence.^53^ The RED software was used to calculate the partial atomic charges for the modified ^F^rG residues for subsequent calculations with Amber16.^54^, ^55^ A restrained simulated annealing was performed in implicit water using the parmbsc0 force field including the χ_OL4_, *ϵ*ζ_OL1_, and β_OL1_ corrections.^56^, ^57^, ^58^, ^59^ Twenty lowest‐energy structures were selected, equilibrated for 1 ns with explicit solvent, and shortly minimized in vacuum. Atomic coordinates of an ensemble of ten final structures with the lowest energy have been deposited in the Protein Data Bank (accession code 6RS3). Details of the calculation process can be found in the Supporting Information. Structural parameters were determined with the 3DNA software package.^60^

### Accession codes

Atomic coordinates of the F(14,15) G‐quadruplex have been deposited in the Protein Data Bank (accession code 6RS3).

## Conflict of interest

The authors declare no conflict of interest.

## Supporting information

As a service to our authors and readers, this journal provides supporting information supplied by the authors. Such materials are peer reviewed and may be re‐organized for online delivery, but are not copy‐edited or typeset. Technical support issues arising from supporting information (other than missing files) should be addressed to the authors.

Supplementary
