# Supplementary material for: Sugar Puckering Drives G‐Quadruplex Refolding: Implications for V‐Shaped Loops
Source: Chemistry. 2019 Dec 10;26(2):524–33. doi: 10.1002/chem.201904044 (PMC6973071; doi:10.1002/chem.201904044)
Supplement: Supplementary file 1 — Supplementary [file CHEM-26-524-s001.pdf]

CHEMISTRY
A European Journal

Supporting Information

Sugar Puckering Drives G-Quadruplex Refolding: Implications for V-Shaped Loops

Linn Haase,[a] Jonathan Dickerhoff,[a, b] and Klaus Weisz*[a]

chem_201904044_sm_miscellaneous_information.pdf

Author Contributions

L.H. Conceptualization: Equal; Formal analysis: Lead; Investigation: Lead; Methodology: Equal; Validation: Lead; Writing - Original Draft: Lead; Writing - Review & Editing: Equal
J.D. Conceptualization: Equal; Formal analysis: Supporting; Investigation: Supporting; Methodology: Equal; Validation: Supporting; Writing - Review & Editing: Supporting
K.W. Conceptualization: Equal; Formal analysis: Supporting; Funding acquisition: Lead; Methodology: Equal; Project administration: Lead; Validation: Supporting; Writing - Original Draft: Supporting; Writing - Review & Editing: Equal.

Supporting Information

NMR experiments

Spectra were acquired at temperatures between 25 and 40 °C with ^{1}H chemical shifts referenced relative to H_2O. A low-salt 10 mM KP_i buffer was used for a better signal-to-noise ratio despite low melting temperatures of F(14,15) and R(14,15) (see Table S1). Although unfolded species significantly contributed to spectra acquired in low-salt buffer at higher temperatures (> 30 °C), the signal-to-noise ratio was superior when compared to spectra acquired in a high-salt buffer containing 120 mM K^+ (see Figure S18). ^{15}N editing HMQC experiments separately optimized for guanine H1 and H8 detection were acquired with 4K scans on 0.2 mM samples (5-10% ^{15}N enrichment). For H1, selective excitation through a low-power $\pi/2$ shaped pulse with a bandwidth of 1 kHz in the center of the imino region was followed by the standard HMQC sequence with ^{15}N decoupling during acquisition. For H8, a pulse sequence containing ^{15}N π shaped pulses for a selective refocusing of scalar coupling to N9 and a ^{15}N $\pi/2$ purge pulse was used as a 1D version of the originally published 2D experiment.[1] This pulse sequence was not further optimized for the detection of adenosine H2 resonances because of similar offsets for N1 and N3 always leading to considerable signal loss through multiple quantum terms.

A WATERGATE with w5 element was employed for solvent suppression in 1D spectra and 2D NOE experiments. NOESY spectra were recorded with mixing times of 80 to 300 ms in either 90% H_2O/10% D_2O or 100% D_2O. DQF-COSY spectra were acquired in D_2O with a 3-9-19 binomial sequence used for solvent suppression. Phase-sensitive ^{1}H-^{13}C HSQC experiments optimized for a ^{1}J(C,H) of 170 Hz were acquired with a 3-9-19 solvent suppression scheme in 90% H_2O/10% D_2O employing a spectral width of 4.5 kHz in the indirect ^{13}C dimension and 256 t_1 increments. ^{13}C chemical shifts were referenced relative to DSS by using the indirect referencing method.

^{19}F spectra were acquired with a spectral width of 5.5 kHz and 8K data points with and without broadband ^{1}H decoupling using the waltz16 sequence. Selective ^{1}H decoupling was achieved through continuous wave irradiation at the corresponding offset with power levels of 40 dB. 2D ^{19}F-^{1}H HOESY spectra with mixing times of 200 and 350 ms were acquired with a spectral width of 5.9 kHz in the indirect ^{1}H dimension and 600 t_1 increments. ^{19}F chemical shifts were referenced relative to TFA using the indirect referencing method (factor 0.940867).[2]

NMR structure calculations

2D NOE crosspeaks were classified as strong (2.9 ± 1.1 Å), medium (4.0 ± 1.5 Å), weak (5.5 ± 1.5 Å), or very weak (6.0 ± 1.5 Å). For exchangeable protons, categories were set to medium (4.0 ± 1.2 Å), weak (5.0 ± 1.2 Å), or very weak (6.0 ± 1.2 Å). In case of strongly overlapped signals, distances were set to 5.0 ± 2.0 Å. Glycosidic torsion angles were restrained to 170-310° for *anti* conformers. Glycosidic torsions for *syn* conformers were restrained to 25-95° except for ^{F}rG14 for which the range was extended up to 110° for more conformational flexibility. The pseudorotation phase angle was restricted to 144-180° for experimentally determined *south*-type conformers (applies to most DNA

residues based on DQF-COSY spectra) and 35-90° for ^{F}rG15. For a simulated annealing of the 100 starting structures, a 5 ps equilibration period at 300 K was followed by heating to 1000 K during 10 ps. After 30 ps, the system was cooled to 100 K and finally to 0 K within 45 ps and 10 ps, respectively. Force constants for NMR-derived distance restraints were set to 40 kcal·mol^{-1}·Å^{-2}, for hydrogen bond restraints to 50 kcal·mol^{-1}·Å^{-2}, for glycosidic torsion angle and sugar pucker restraints to 200 kcal·mol^{-1}·rad^{-2}, and for planarity restraints of G-tetrads to 30 kcal·mol^{-1}·Å^{-2}. For a refinement in water, the quadruplexes were neutralized with K^+ ions and two of the cations placed in the center between the eight O6 atoms of two adjacent tetrads. The system was hydrated with TIP3P water molecules in a truncated octahedral box of 10 Å.[3] During initial equilibration, the DNA was fixed with 25 kcal·mol^{-1}·Å^{-2}. After 500 steps of steepest descent and conjugate gradient minimization, the system was heated from 100 to 300 K during 10 ps under constant volume, followed by a decrease in force constants to 5, 4, 3, 2, 1, and 0.5 kcal·mol^{-1}·Å^{-2} and further equilibration. The final simulation of 1 ns duration at 1 atm was performed with restraints only for NMR-derived distances and Hoogsteen hydrogen bonds. The trajectories were subsequently averaged over the last 100 ps and shortly minimized in vacuum with 500 steps of steepest descent, followed by 500 steps of conjugate gradient minimization.

References

[1] A. T. Phan, D. J. Patel, *J. Am. Chem. Soc.* **2002**, *124*, 1160–1161.

[2] T. Maurer, H. R. Kalbitzer, *J. Magn. Reson. Ser. B* **1996**, *113*, 177–178.

[3] W. L. Jorgensen, J. Chandrasekhar, J. D. Madura, R. W. Impey, M. L. Klein, *J. Chem. Phys.* **1983**, *79*, 926–935.

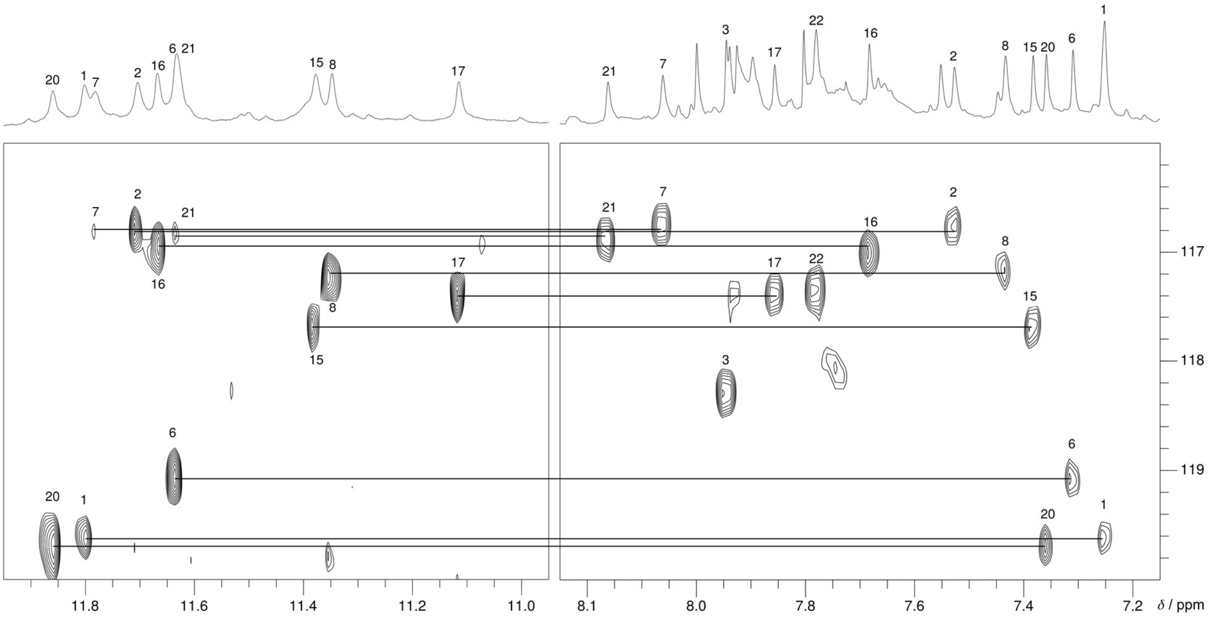

Figure S1. ^{1}H-^{13}C HMBC spectrum of F(14,15) (1 mM) acquired at 25 °C in 10 mM KP_i, pH 7, correlating guanine H1 and H8 protons to ^{13}C5.

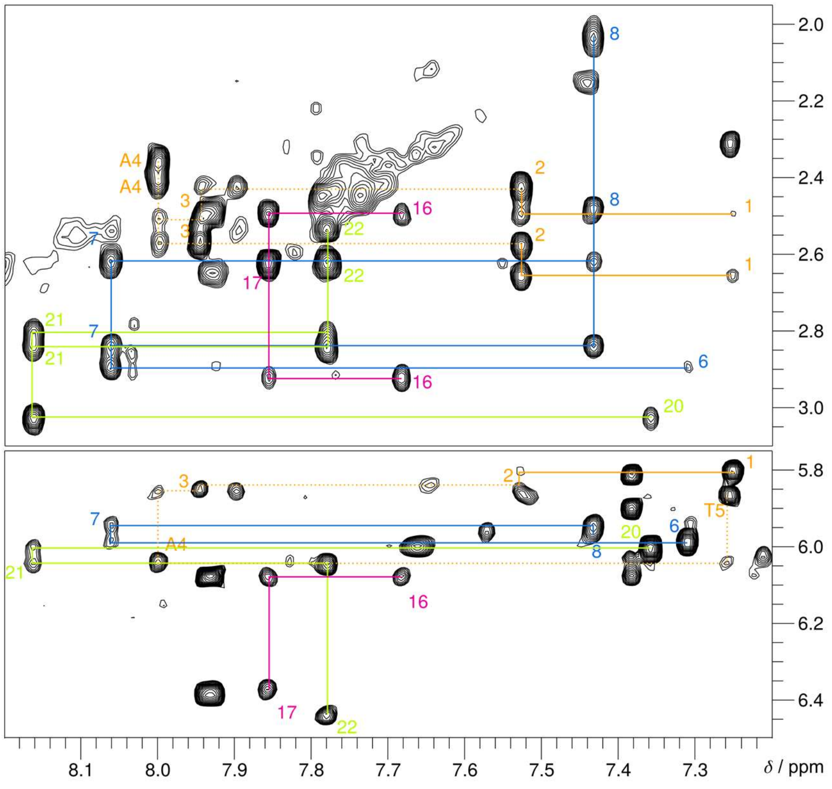

Figure S2. Portions of a 2D NOE spectrum of F(14,15) (1 mM) acquired at 25 °C in 10 mM KP_i, pH 7. Sequential contacts are traced in different colors for the four G-tracts in the aromatic-H2' (top) and the aromatic-H1' region (bottom). Dashed lines indicate sequential contacts extending into loop regions.

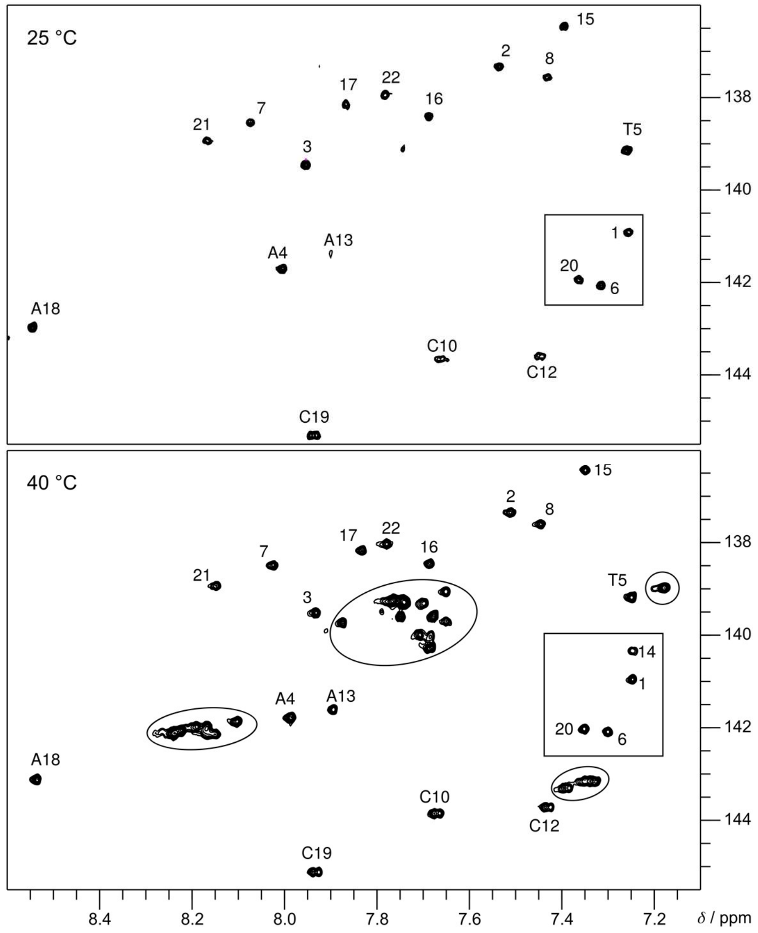

Figure S3. ^{1}H-^{13}C HSQC spectra (H6/8-C6/8 region) of F(14,15) (1 mM) acquired in 10 mM KP_i buffer, pH 7, at 25 °C (top) and 40 °C (bottom). Correlations from s*yn* guanosines and from unfolded species are framed by rectangles and ellipses, respectively. Note that ^{F}rG14 is only observed at 40 °C, justifying acquisition of spectra for topology and structure determination at elevated temperatures in spite of partial unfolding (see Table S1).

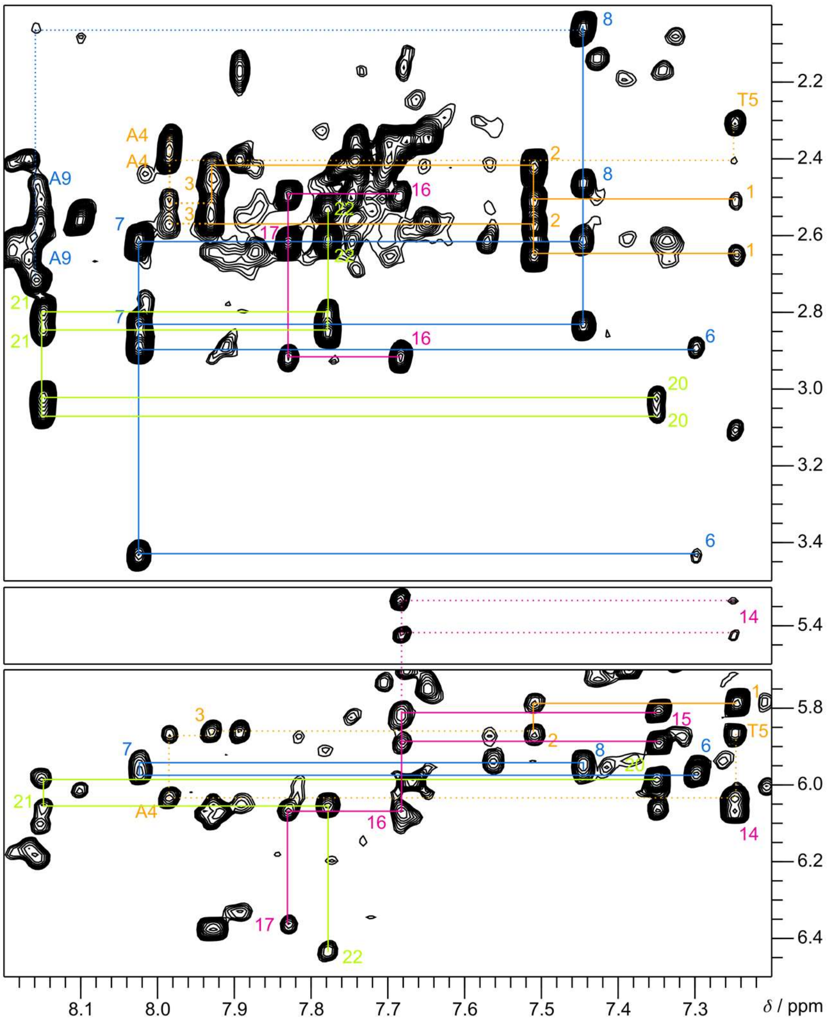

Figure S4. Regions of a 2D NOE spectrum of F(14,15) (1 mM) acquired at 40 °C in 10 mM KP_i, pH 7. Sequential contacts are traced in different colors for the four G-tracts in the aromatic-H2' (top) and the aromatic-H1' region (bottom). The middle panel shows the weak H8-H2' contact of ^{F}rG14 in line with its *syn* conformation and the unusual contact of G16 H8 to ^{F}rG14 H2'. Dashed lines indicate sequential contacts extending into loop regions.

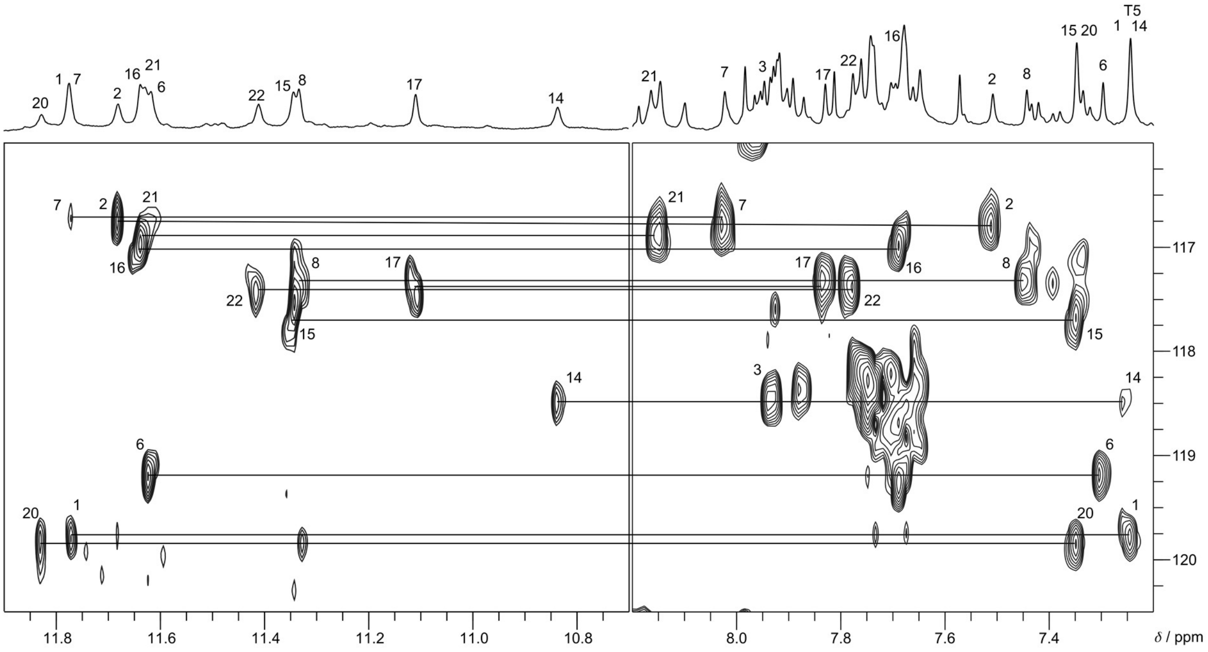

Figure S5. ^{1}H-^{13}C HMBC spectrum of F(14,15) (1 mM) acquired at 40 °C in 10 mM KP_i, pH 7, correlating guanine H1 and H8 protons to $^{13}C5$.

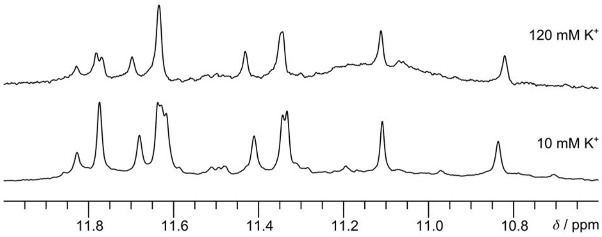

Figure S6. Comparison of the imino proton spectral region of F(14,15) (1 mM) acquired at 40 °C in a low-salt (10 mM KP_i, pH 7) and a high-salt buffer (20 mM KP_i, 100 mM KCl, pH 7). Note how the signal to noise ratio in low-salt buffer is superior in spite of partial quadruplex unfolding as already observed under these conditions.

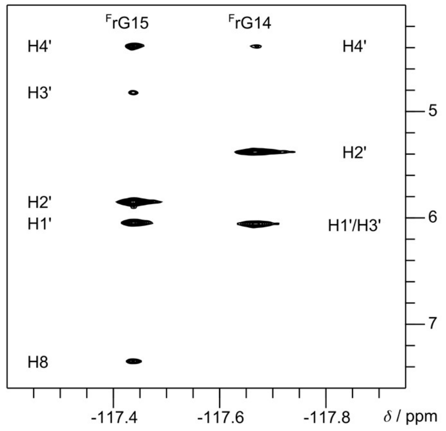

Figure S7. 2D ^{19}F-^{1}H HOESY spectrum (mixing time 350 ms) of F(14,15) (1 mM) acquired at 40 °C in 10 mM KP_i, pH 7. F2' resonances of ^{F}rG14 and ^{F}rG15 are identified by correlations to sugar protons. In contrast to *syn* ^{F}rG14, *anti* ^{F}rG15 additionally exhibits an intranucleotide F2'-H8 correlation.

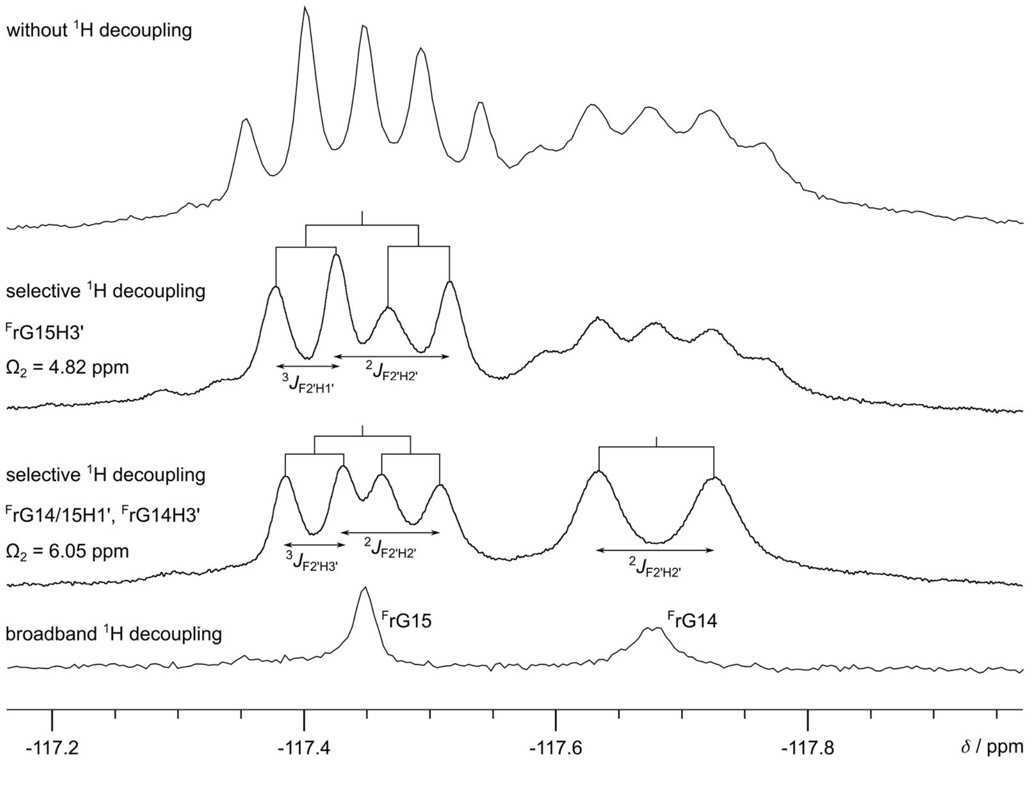

Figure S8. ^{19}F spectra of F(14,15) (1 mM) acquired at 40 °C in 10 mM KP_i, pH 7, without ^{1}H decoupling (top spectrum), with selective decoupling of H1' and H3' resonances (two middle spectra) and with broadband ^{1}H decoupling (bottom spectrum). $^{3}J_{F2'H1'}$ and $^{3}J_{F2'H3'}$ of ^{F}rG15 are determined from the doublet of doublet under selective decoupling of the H3' and H1' resonance, respectively. Note that $^{2}J_{F2'H2'}$ of ^{F}rG15 extracted under selective H1' decoupling is too small due to additional partial H2' decoupling (difference of H1' and H2' chemical shifts only amounts to 0.2 ppm). For ^{F}rG14, only $^{2}J_{F2'H2'}$ can be determined because H1' and H3' are almost isochronous.

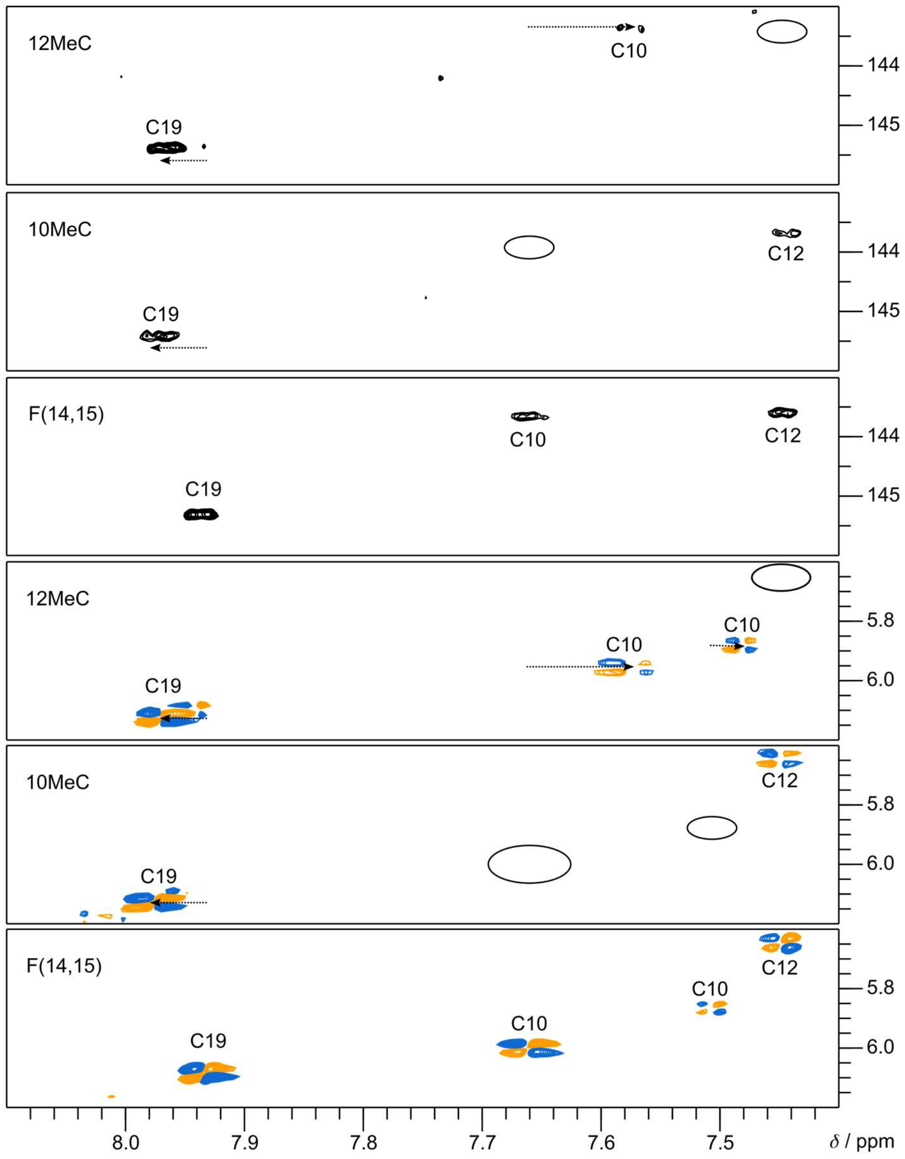

Figure S9. Assignment of F(14,15) cytidine resonances through individual 5-methyl-dC labeling at position 10 and 12. H6-C6 region of ^{1}H-^{13}C HSQC spectra (top) and H5-H6 region of DQF-COSY spectra (bottom) for unlabeled and labeled F(14,15) in 10 mM KP_i at 25 °C, pH 7. H6-C6 HSQC correlations for labeled positions disappear from the typical spectral region due to significant upfield shifts as a consequence of the C5 methyl substituent; H6-H5 correlations are lost in DQF-COSY spectra for 5-methyl-dC. Note that a less populated conformer within the long lateral loop gives rise to an additional C10 DQF-COSY crosspeak. Positions of lost and slightly shifted signals for labeled samples are indicated by ellipses and arrows, respectively.

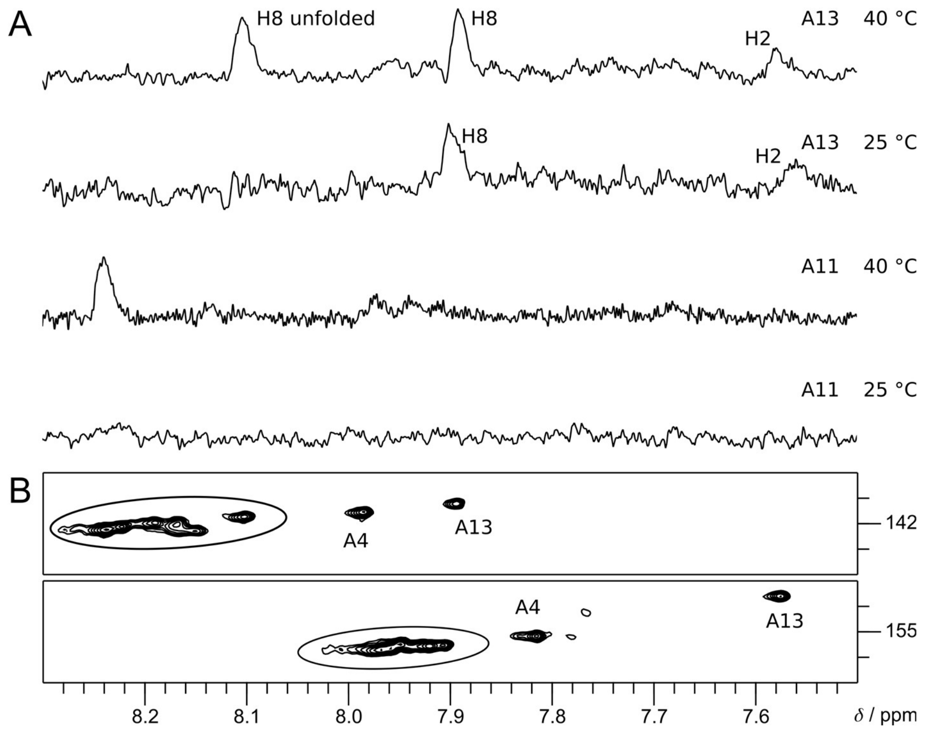

Figure S10. Assignment of F(14,15) adenosine resonances through individual ^{15}N-dA labeling at position 11 and 13. (A) One-dimensional ^{1}H-^{15}N HMBC spectra acquired at 25 °C and 40 °C with typical concentrations of 0.2 mM in 10 mM KP_i buffer, pH 7. In addition to an H8 signal arising from unfolded species, H8 and H2 resonances are clearly observable for A13 at 40 °C; resonance assignments for A11 are ambiguous due to possible overlap with signals from unfolded species at 40 °C whereas no signals are observed at 25 °C. (B) Regions of a ^{1}H-^{13}C HSQC spectrum acquired at 40 °C in 10 mM KP_i, pH 7, showing H8-C8 (top) and H2-C2 (bottom) correlations of F(14,15) (1 mM). Clusters of signals primarily from unfolded species are framed by ellipses. Note, that the ^{1}H chemical shift scale in ω_2 also applies to the 1D spectra in (A).

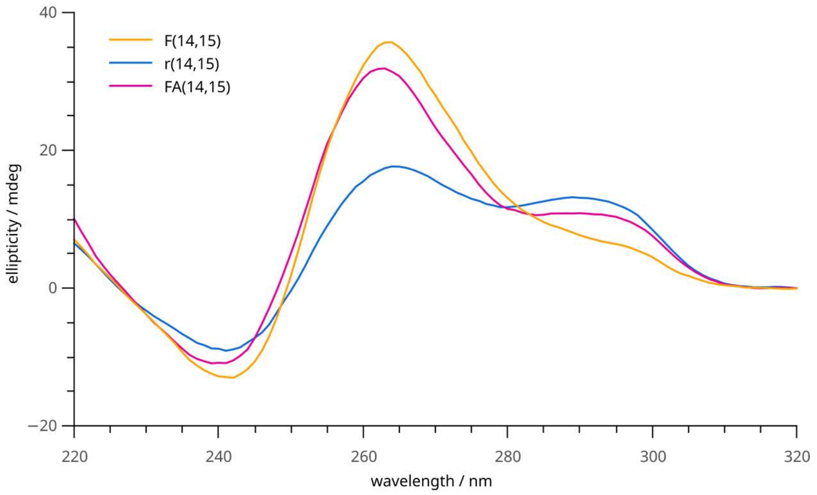

Figure S11. CD spectra of 14,15-modified *ODN*. Spectra were recorded at 20 °C in 20 mM KP_i buffer containing 100 mM KCl, pH 7. All three modifications exhibit one negative band around 240 nm along with two positive contributions of varied amplitudes at 260 nm and 290 nm, typical of a hybrid-type quadruplex with both homopolar and heteropolar stacking interactions.

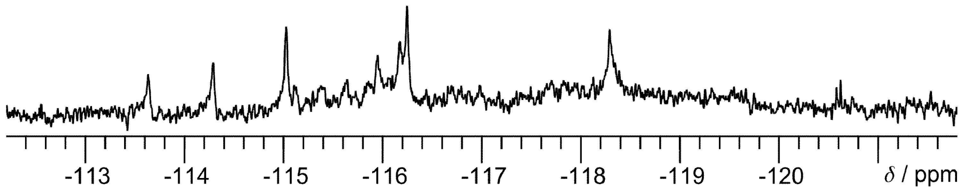

Figure S12. ^{1}H decoupled ^{19}F spectrum of FA(14,15) (0.3 mM) acquired at 25 °C in 10 mM KP_i, pH 7. The presence of at least six distinct resonances suggests three or more coexisting species.

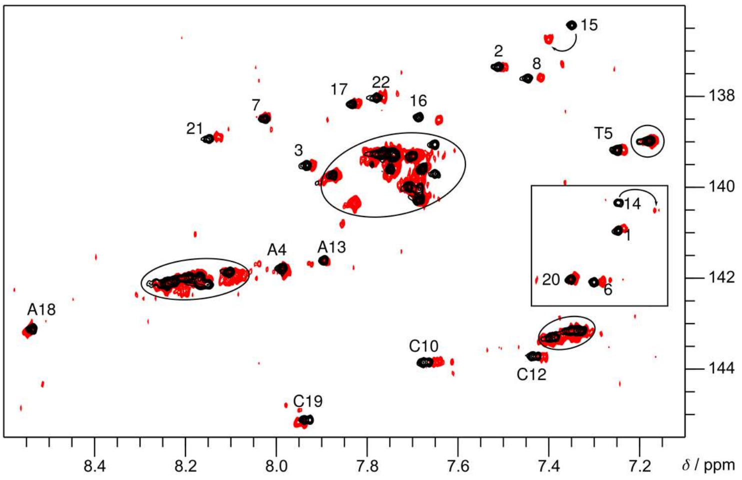

Figure S13. Superimposed H6/8-C6/8 regions from ^{1}H-^{13}C HSQC spectra of r(14,15) (0.5 mM, red) and F(14,15) (1 mM, black) at 40 °C in 10 mM KP_i buffer, pH 7. S*yn* guanosines including ^{F}rG14/rG14 and signals arising from unfolded species are framed by rectangles and ellipses, respectively. The poorer spectral quality for r(14,15) can be attributed to its lower concentration and higher degree of unfolding due to a lower thermal stability (Table S1). Nevertheless, the majority of signals exhibit an almost perfect overlap with slight shifts experienced by resonances in the modified positions 14 and 15 (indicated by arrows).

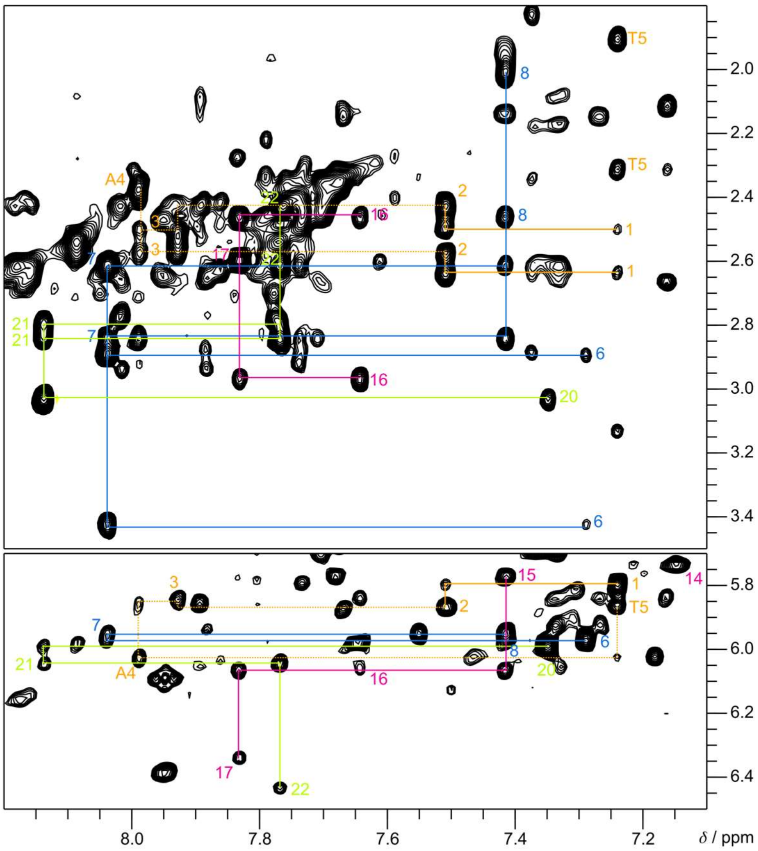

Figure S14. Portions of a 2D NOE spectrum of r(14,15) (0.5 mM) acquired at 35 °C in 10 mM KP_i, pH 7: Sequential contacts are traced in different colors for the four G-tracts in the aromatic-H2' (top) and the aromatic-H1' region (bottom). Dashed lines indicate sequential contacts extending into loop regions.

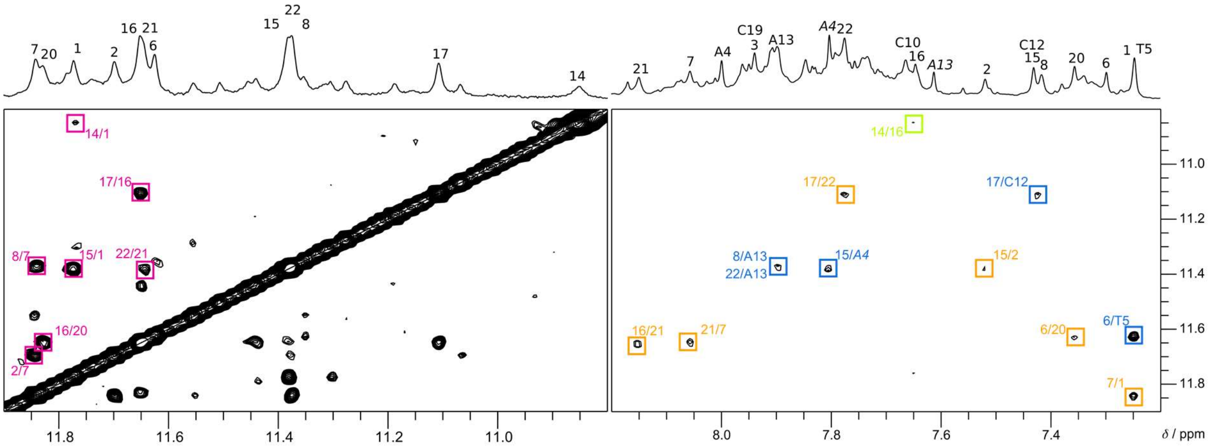

Figure S15. Regions of a 2D NOE spectrum of r(14,15) (0.5 mM) acquired at 30 °C in 10 mM KP_i, pH 7. Inter-tetrad H1(ω_1)-H1(ω_2) contacts indicative of relative tetrad polarities (framed in purple) and intra-tetrad H8(ω_1)-H1(ω_2) contacts reflecting the hydrogen bond directionality within tetrads (framed in orange) are labeled together with additional H8-H1 NOE contacts between tetrads (framed in green) and from outer tetrad imino resonances to aromatic protons of residues in the two lateral loops (framed in blue), notably A4, T5, A13, and C12. Adenosine H2 resonances are labeled in italic.

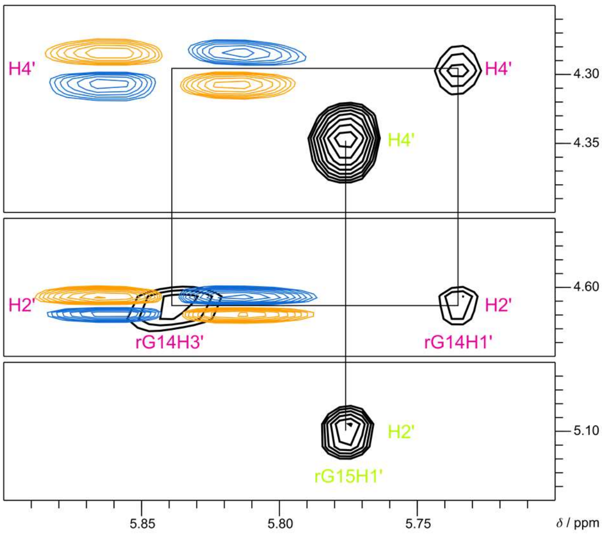

Figure S16. Portions of a 2D NOE spectrum (black) superimposed onto a DQF-COSY spectrum recorded in D_2O (colored) of r(14,15) (0.5 mM). Spectra were acquired at 35 °C in 10 mM KP_i, pH 7, and show correlations within the furanose ring. The absence of H2'(ω_1)-H1'(ω_2) correlations in the DQF-COSY spectrum in striking contrast to clearly observable H2'-H1' NOE contacts (labeled in purple and green in the middle and bottom panel, respectively) point to a *north*-type sugar for both rG14 and rG15. The strong H4'(ω_1)-H1'(ω_2) NOE contact for rG15 (top panel) implies a *north/north-east* pucker. The unusually downfield shifted H3' resonance of rG14 is identified by DQF-COSY crosspeaks with H2' (middle panel) and H4' (top panel).

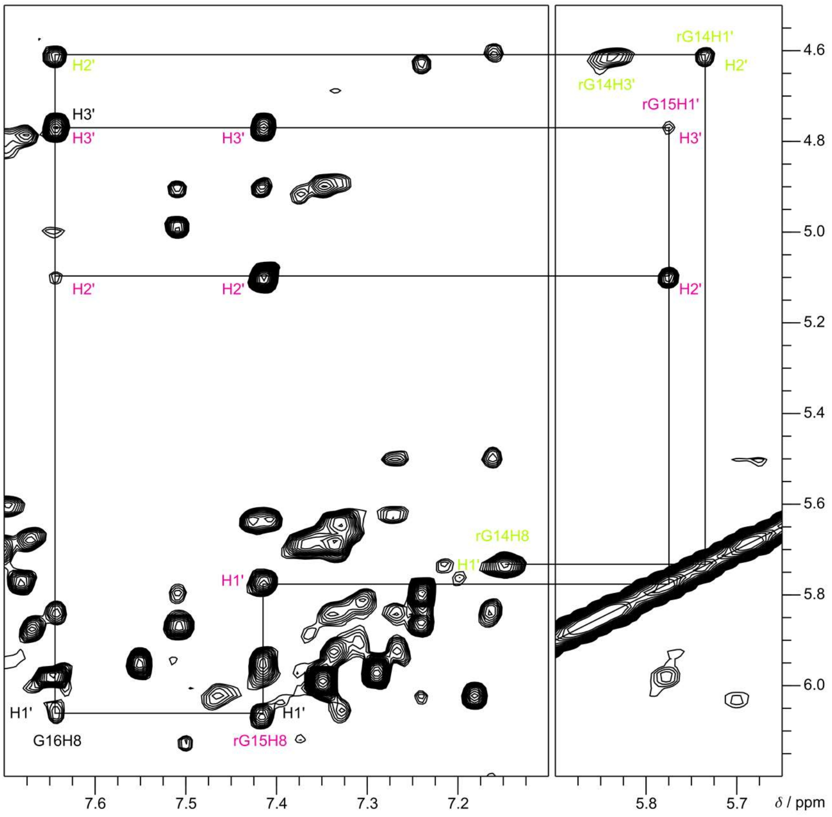

Figure S17. Regions of a 2D NOE spectrum of r(14,15) (0.5 mM) acquired at 35 °C in 10 mM KP_i, pH 7, showing intra- and internucleotide contacts of V-loop flanking residues rG14 (in green) and rG15 (in purple) as well as of G16 (in black). Note the unusual $H8_i$-$H1'_{i+1}$ (rG15 H8 - G16 H1') and $H8_i$-$H2'_{i-2}$ (G16 H8 - rG14 H2') NOE crosspeak.

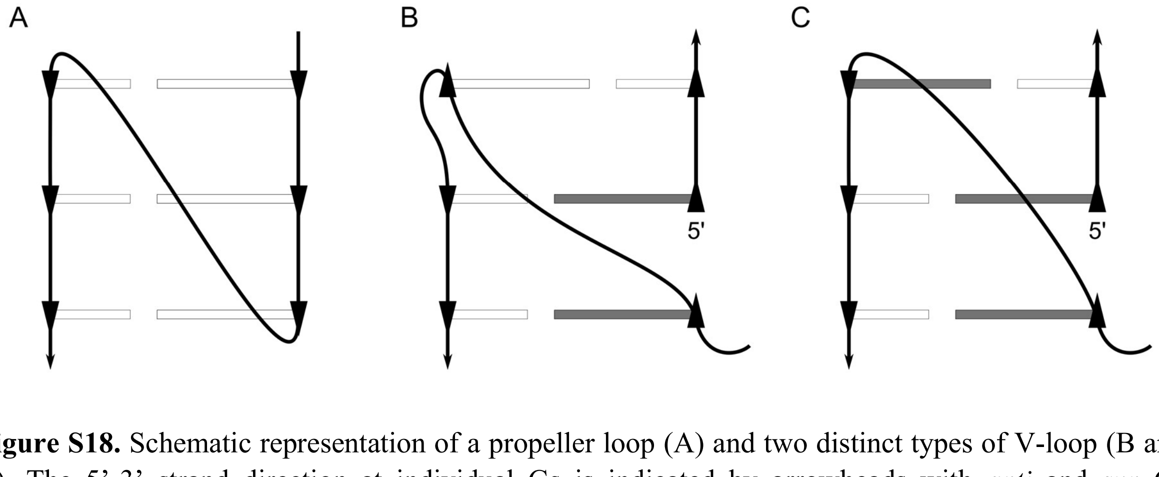

Figure S18. Schematic representation of a propeller loop (A) and two distinct types of V-loop (B and C). The 5'-3' strand direction at individual Gs is indicated by arrowheads with *anti* and *syn* Gs represented by white and grey rectangles, respectively. In contrast to the propeller loop connecting parallel strands, a V-loop links two antiparallel G-tracts. It generally starts at the bottom of an interrupted G-tract with the 5'-terminal G mostly located in the central tetrad. Of note, the orientation of the V-loop 5'-flanking G is upward in contrast a G preceding a propeller loop. The V-loops depicted in (B) and (C) differ in the 5'-3' orientation of the V-loop 3'-flanking G. It is turned upward (B) or downward (C) associated with an *anti* or *syn* glycosidic torsion angle and a strand polarity inversion after or within the V-loop, respectively.

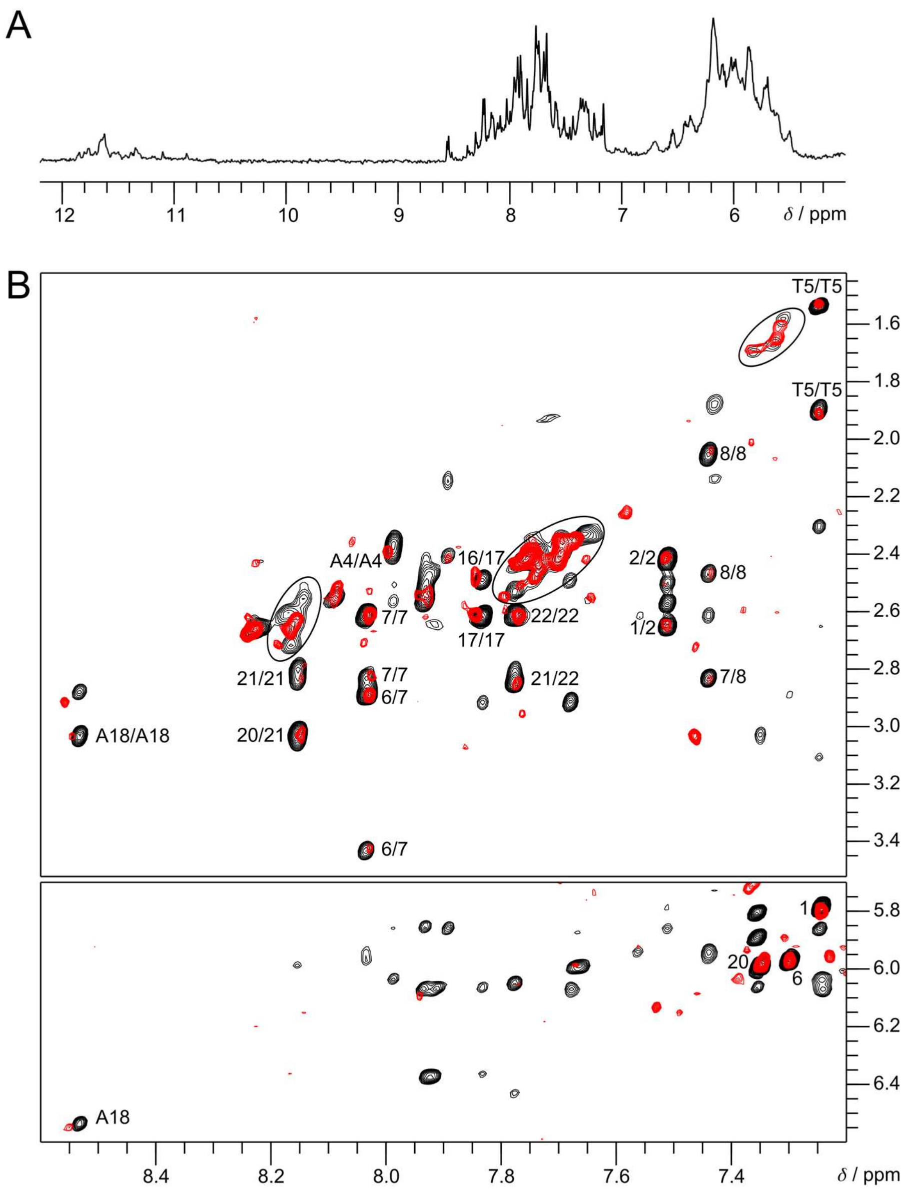

Figure S19. (A) One-dimensional ^{1}H spectrum of F(14) and (B) 2D NOE spectra of F(14) (red) and F(14,15) (black) acquired at 35 °C in 10 mM KP_i buffer, pH 7. The presence of only very weak imino signals in the 10-12 ppm region as shown in (A) indicates only partial quadruplex formation. (B) A superposition of 2D NOE spectra for F(14) and F(14,15) in the H2'/H2''(ω_1)-H8/H6(ω_2) (top) and H1'(ω_1)-H8/H6(ω_2) region reveals almost perfect overlap between most of the few F(14) characteristic crosspeaks and high-intensity NOE contacts of F(14,15). Signals arising from unfolded species are framed by ellipses.

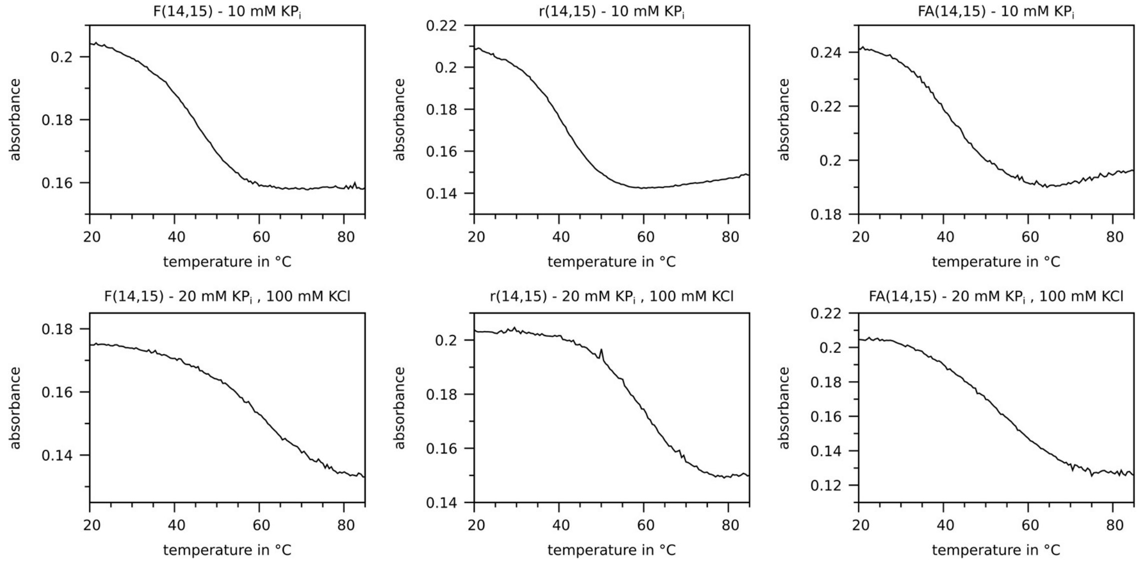

Figure S20. Representative UV-melting curves (heating phase) of F(14,15), r(14,15), and FA(14,15) (5 µM) in 10 mM KP_i, pH 7 (top) and 20 mM KP_i, 100 mM KCl, pH 7 (bottom). The absorbance at 295 nm is plotted as a function of temperature.

Table S1. UV melting temperatures (T_m)[a] of 14,15-disubstituted *ODN* sequences in low-salt and high-salt potassium buffer, pH 7.

T_m / °C	10 mM KP_i	20 mM KP_i, 100 mM KCl
F(14,15)	45.1 ± 0.5	61.1 ± 0.3
r(14,15)	41.0 ± 0.7	59.1 ± 0.6
FA(14,15)	40.0 ± 1.4	54.9 ± 0.5

[a] measured in triplicate

Table S2. Experimentally determined $^3J_{HF}$ scalar couplings of ^{F}rG14 and ^{F}rG15 in F(14,15) at 40 °C.[a]

	^{F}rG14	^{F}rG15
^2J(F2‘,H2‘)	52.1 Hz[b] / 52.8 Hz[c]	50.8 Hz[b] / 51.0 Hz[c]
^3J(F2‘,H1‘)	24.0 Hz[c]	27.8 Hz[b] / 28.2 Hz[c]
^3J(F2‘,H3‘)	27.0 Hz[c]	26.5 Hz[b] / 27.0 Hz[c]

[a] uncertainty ± 1 Hz
[b] from ^{19}F spectra with selective ^{1}H decoupling
[c] from ^{1}H-^{1}H 2D NOE and DQF-COSY crosspeaks

Table S3. NMR restraints and structural statistics for the structure calculations of F(14,15).

**NOE distance restraints**	
intraresidual	80
sequential	67
long range	45
**other restraints**	
hydrogen bonds	48
torsion angles	39
**structural statistics**	
pairwise heavy atom RMSD / Å	
G-core	0.84 ± 0.15
all residues	2.96 ± 0.71
violations / Å	
maximum NOE violation	0.25
mean NOE violation	0.005 ± 0.002
deviations from idealized geometry	
bonds / Å	0.01 ± 0.00
angles / degree	2.40 ± 0.03

Table S4. ^{1}H, ^{13}C, and ^{19}F chemical shifts of F(14,15) at 40 °C in 10 mM KP_i, pH 7.[a]

	H6/H8	C6/C8	C5	H1'	H2'/H2"	H3'	H4'	H1	H5/H2/Me/ F2'
G1	7.24	140.9	119.8	5.79	2.51/2.65	4.90	n.d.	11.77	-
G2	7.51	137.4	116.8	5.87	2.42/2.57	4.99	n.d.	11.68	-
G3	7.93	139.5	118.5	5.87	2.51/2.57[b]	4.83	n.d.	n.d.	-
A4	7.99	141.8	n.d.	6.03	2.37/2.41	4.75	n.d.	-	7.82
T5	7.25	139.2	n.d.	5.86	1.90/2.31	n.d.	n.d.	n.d.	1.53
G6	7.30	142.1	119.1	5.97	3.43/2.89	4.86	n.d.	11.62	-
G7	8.02	138.5	116.7	5.94	2.61/2.83	5.04	n.d.	11.77	-
G8	7.44	137.6	117.3	5.97	2.06/2.47	4.90	n.d.	11.33	-
A9	8.16	n.d.	n.d.	6.19	2.47/2.72[b]	4.97	n.d.	-	n.d.
C10	7.67	143.8	n.d.	6.08	1.87/2.24	4.89	n.d.	-	5.99
A11	n.d.	n.d.	n.d.	n.d.	n.d.	n.d.	n.d.	-	n.d.
C12	7.43	143.7	n.d.	5.73	1.87/2.14	4.53	n.d.	-	5.67
A13	7.89	141.6	n.d.	5.85	2.17/2.41[b]	4.81	n.d.	-	7.56
^{F}rG14	7.25	140.3	118.5	6.05	5.38	6.09	4.38	10.84	-117.67
^{F}rG15	7.35	136.4	117.7	6.04	5.85	4.82	4.38	11.35	-117.44
G16	7.68	138.5	117.0	6.07	2.92/2.50	4.78	n.d.	11.64	-
G17	7.83	138.2	117.3	6.36	2.62/2.62	5.04	n.d.	11.11	-
A18	8.53	143.1	n.d.	6.54	3.04/2.88	5.01	n.d.	-	8.31
C19	7.93	145.1	n.d.	6.38	2.46/2.65[b]	4.93	n.d.	-	6.07
G20	7.35	142.0	119.9	5.99	3.06/3.02	4.98	n.d.	11.83	-
G21	8.15	138.9	116.9	6.05	2.80/2.85	5.10	n.d.	11.63	-
G22	7.78	138.0	117.4	6.43	2.61/2.53	4.73	n.d.	11.41	-

[a] nd: not determined
[b] no stereospecific assignments

Table S5. ^{1}H and ^{13}C chemical shifts of r(14,15) at 35 °C in 10 mM KP_i, pH 7.[a]

res	H8/H6	C8/C6	H1'	H2'/H2''[b]	H3'	H4'	H1	H5/H2/Me
G1	7.24	140.9	5.80	2.49/2.64	4.90	n.d.	11.76	-
G2	7.51	137.4	5.86	2.43/2.58	4.99	n.d.	11.68	-
G3	7.93	139.5	5.84	2.50/2.58	4.83	n.d.	n.d.	-
A4	7.99	141.8	6.03	2.38/2.38	4.75	n.d.	-	7.80
T5	7.24	139.1	5.86	1.90/2.31	n.d.	n.d.	n.d.	1.53
G6	7.29	142.1	5.97	2.89/3.42	4.48	n.d.	11.61	-
G7	8.04	138.5	5.95	2.62/2.84	5.04	n.d.	11.83	-
G8	7.41	137.6	5.97	2.01/2.46	4.90	n.d.	11.37	-
A9	n.d.	n.d.	n.d.	n.d./n.d.	n.d.	n.d.	-	n.d.
C10	7.65	143.8	n.d.	n.d./n.d.	n.d.	n.d.	-	5.98
A11	n.d.	n.d.	n.d.	n.d./n.d.	n.d.	n.d.	-	n.d.
C12	7.42	143.7	5.77	1.94/2.14	n.d.	n.d.	-	5.64
A13	7.89	141.6	5.85	2.09/2.43	n.d.	n.d.	-	7.62
rG14	7.15	140.4	5.73	4.61/ -	5.84	4.29	10.83	-
rG15	7.41	136.8	5.77	5.10/ -	4.77	4.35	11.37	-
G16	7.64	138.5	6.06	2.46/2.97	4.78	n.d.	11.64	-
G17	7.83	138.2	6.34	2.60/2.60	5.03	n.d.	11.10	-
A18	8.55	143.1	6.54	2.89/3.04	4.82	n.d.	-	8.32
C19	7.95	145.2	6.39	2.48/2.65	n.d.	n.d.	-	6.09
G20	7.35	142.0	5.99	3.03/3.03	4.90	n.d.	11.81	-
G21	8.14	138.9	6.04	2.80/2.84	5.10	n.d.	11.64	-
G22	7.77	138.0	6.43	2.54/2.61	4.72	n.d.	11.38	-

[a] nd: not determined
[b] no stereospecific assignments
